# Elucidating the Lipid Binding Properties of Membrane-Active Peptides Using Cyclised Nanodiscs

**DOI:** 10.3389/fchem.2019.00238

**Published:** 2019-04-16

**Authors:** Alan H. Zhang, Ingrid A. Edwards, Biswa P. Mishra, Gagan Sharma, Michael D. Healy, Alysha G. Elliott, Mark A. T. Blaskovich, Matthew A. Cooper, Brett M. Collins, Xinying Jia, Mehdi Mobli

**Affiliations:** ^1^Centre for Advanced Imaging, The University of Queensland, Brisbane, QLD, Australia; ^2^Institute for Molecular Bioscience, The University of Queensland, Brisbane, QLD, Australia

**Keywords:** arenicin, VSTx1, nanodiscs, POPC, POPG, cNW9, membrane-active peptide

## Abstract

The lipid composition of the cellular membrane plays an important role in a number of biological processes including the binding of membrane-active peptides. Characterization of membrane binding remains challenging, due to the technical limitations associated with the use of standard biophysical techniques and available membrane models. Here, we investigate the lipid binding properties of two membrane-active peptides, VSTx1, a well characterized ion-channel inhibitor, identified from spider venom, that preferentially binds to anionic lipid mixtures, and AA139 an antimicrobial β-hairpin peptide with uncharacterised lipid binding properties, currently in pre-clinical development. The lipid binding properties of these peptides are elucidated using nanodiscs formed by both linear and circularized (sortase-mediated) forms of a membrane scaffold protein (MSP1D1ΔH5). We find that nanodiscs formed by circularized MSPs—in contrast to those formed by linear MSPs—are sufficiently stable under sample conditions typically used for biophysical measurements (including lipid composition, a range of buffers, temperatures and concentrations). Using these circularized nanodiscs, we are able to extract detailed thermodynamic data using isothermal titration calorimetry (ITC) as well as atomic resolution mapping of the lipid binding interfaces of our isotope labeled peptides using solution-state, heteronuclear, nuclear magnetic resonance (NMR) spectroscopy. This represents a novel and general approach for elucidating the thermodynamics and molecular interface of membrane-active peptides toward flat lipid bilayers of variable composition. Our approach is validated by first determining the thermodynamic parameters and binding interface of VSTx1 toward the lipid bilayer, which shows good agreement with previous studies using lipid micelles and liposomes. The method is then applied to AA139, where the membrane binding properties are unknown. This characterization, involved solving the high-resolution structure of AA139 in solution using NMR spectroscopy and the development of a suitable expression system for isotope labeling. AA139 was found to bind exclusively to anionic membranes with moderate affinity (*K*_d_~low μM), and was found to have a lipid binding interface involving the termini of the β-hairpin structure. The preference of AA139 for anionic lipids supports a role for membrane binding in the mode-of-action of this peptide, which is also consistent with its higher inhibitory activity against bacterial cells compared to mammalian cells. The described approach is a powerful method for investigation of the membrane binding properties of this important class of molecules.

## Introduction

The composition of the lipid bilayer can have a significant impact on a number of biological processes including the trafficking of soluble proteins, the structure, dynamics and function of integral membrane proteins and the action of membrane-active peptides (Escriba et al., 2008). The latter have emerged as an important class of molecules in the search for novel antimicrobials and ion-channel inhibitors (Zhang et al., 2018). A major limitation in the characterization of membrane-active peptides is the lack of detailed thermodynamic, kinetic and structural information regarding their lipid interactions. Such information can usually be facilitated by biophysical studies in solution (Lee, 2018). Structural characterization of peptides is traditionally conducted using nuclear magnetic resonance (NMR) spectroscopy (Klint et al., 2013), while membrane-binding assays are most commonly conducted using a combinations of chromatography and fluorescence methods (Deuis et al., 2016) or surface plasmon resonance analysis (Hodnik and Anderluh, 2013). Thus, structural details of the binding of membrane-active peptides to lipid bilayers is often only available at very low resolution, hampering efforts to elucidate their mode-of-action. High-resolution structural data is often difficult to obtain by X-ray diffraction methods due to difficulties in crystallization of peptide-lipid complexes. Solid-state NMR is often the only method to study these systems at atomic resolution, although the analysis of such data is often challenging and time-consuming (Mani et al., 2006). Solution-state NMR experiments can with relative ease provide high-resolution information about binding interfaces, although such studies have been limited to use of detergent and/or lipid micelles or bicelles as membrane mimetics (Warschawski et al., 2011; Lau et al., 2016). These models do not accurately reflect the geometry of the cell membrane, in particular they have a significantly higher curvature, which can cause peptides to adopt non-native conformations, or to aggregate (Catoire et al., 2014).

Lipid bilayer nanodiscs (ND) have been developed to solubilize and reconstitute membrane proteins in lipid bilayers, and their use is rapidly expanding (Bayburt et al., 2002; Shaw et al., 2004). NDs are self-assembled soluble particles (from ~10 to 50 nm in diameter), where each particle consists of two membrane scaffold proteins (MSPs), derived from human apo-lipoprotein I that is wrapped around a flat phospholipid bilayer. NDs are increasingly being used in biophysical studies, typically for structural studies of membrane proteins and binding interaction studies of lipophilic ligands or ligands that interact with membrane proteins (Hagn et al., 2013; Shenkarev et al., 2014). Recently, a variant of NDs has been described where the *N*- and *C*-termini are ligated via a peptide bond using sortase enzymes (Nasr et al., 2017; Yusuf et al., 2018)–circularized nanodiscs referred to as cNDs hereafter.

A significant advantage of (c)NDs is in their modularity. Different compositions of synthetic phospholipids can be encapsulated, and these soluble discs can then be studied under a range of solution conditions (pH, temperature, salt etc.). It is often possible to incorporate specific lipid compositions and obtain homogeneous NDs (Lee et al., 2015; Yeh et al., 2016). In particular, we are here interested in studying the binding of membrane-active peptides that have shown activity against anionic lipid bilayers mimicking those of Gram-negative bacteria. We employ heteronuclear NMR experiments using isotope labeled peptides to map the lipid binding interface of these peptides at atomic resolution. In these experiments, the nanodisc is unlabelled and therefore remains undetected. To ensure that these discs remain stable under the conditions of typical NMR experiments we have conducted a detailed study of the solution characteristics and stability of linear and circularized NDs containing mixtures of POPC and POPG.

The stability of the NDs were evaluated by size-exclusion chromatography (SEC), electron microscopy (EM) and mass spectrometry (MS), under different storage conditions with variable buffer (pH), temperature and concentration. While non-circularized NDs containing anionic lipids were shown to be poorly stable, excellent stability was found when using the cNDs. The latter was then used for solution-state biophysical studies to investigate the lipid binding interactions of two peptides that are known to exert their function in anionic lipid bilayer environments.

To determine the binding of these membrane-active peptides to model membranes of Gram-negative bacteria in NDs, we first studied the well characterized spider toxin, VSTx1, isolated from the venom of *Grammostola spatulata*. In previous studies using centrifugation coupled with chromatography, the peptide has been shown to partition into liposomes containing mixtures of POPC and POPG, but these studies did not find partitioning of the peptide into liposomes containing only zwitterionic POPC lipids (Jung et al., 2005; Ozawa et al., 2015; Lau et al., 2016). The peptide was, however, found to bind to zwitterionic DHPC (and DM) micelles in NMR studies (Lee and Mackinnon, 2004; Wang et al., 2018).

Here, we first measured the thermodynamics of the binding of the peptide against cNDs using isothermal titrations calorimetry (ITC) experiments. We then mapped the lipid binding interface of the peptide using chemical shift mapping experiments by analysis of, 2D ^1^H-^15^N-HSQC, solution-state NMR experiments. Our results show very weak binding of the peptide to POPC bilayers in cNDs–much weaker than that observed when using DHPC micelles. In contrast, the peptide binds very strongly to anionic cNDs, consistent with the previous liposome experiments, suggesting that the binding of the peptide to micelles is different than to bilayers.

Next, the described approach was applied to an antimicrobial peptide, AA139, currently undergoing preclinical trials for the treatment of Gram-negative bacterial infections. AA139 is an analog of arenicin-3, a peptide antibiotic that was originally identified as part of a group of broad-spectrum antimicrobial peptides isolated from the lugworm *Arenicola marina* by *Novozymes* (Novozymes A/S Copenhagen). The mode-of-action of this peptide remains unknown; however, the related arenicin-2 peptide was shown to form pores in planar lipid bilayers, suggesting a cytotoxic mode-of-action (Shenkarev et al., 2011). A bacterial expression system was developed for recombinant expression and labeling of the peptide. To map the binding interface of the peptide, the structure of the peptide was also solved in solution by standard NMR methods, revealing a twisted β-hairpin fold, common in this family of peptides. The biophysical data (EM/NMR/ITC) show that AA139 does bind to anionic cNDs but does not form pores, suggesting a different mode-of-action to arenicin-2 (Shenkarev et al., 2011).

The presented approach provides a platform for measurement of both thermodynamic and structural data for membrane-active peptides using a planar bilayer system, using standard NMR experiments. The flexibility and stability of the cNDs as a model system promises to improve our understanding of this important class of molecules.

## Materials and Methods

### Materials

The construct for the evolved pentamutant of Sortase A in a pET29 vector was a gift from Prof. David R. Liu's laboratory (Harvard University). Expression and purification of the pentamutant Sortase A from *Escherichia coli* BL21(DE3) cells was performed as previously described (Chen et al., 2011). The construct for MSP1D1ΔH5 (dH5 hereafter) in a pET28a vector was a gift from Prof. Gerhard Wagner at the Harvard Medical School. Using NEB Q5 Site-Directed Mutagenesis Kit (New England BioLabs, NEB), a DNA sequence encoding LPGTGAAALEHHHHHH was appended into the end of the encoding sequence for MSP1D1ΔH5 construct to create the NW9 construct. Synthetic lipids, palmitoyloleoyl-phosphatidylcholine (POPC) and palmitoyloleoyl-phosphatidylglycerol (POPG), in powder form were purchased from Avanti Polar Lipids (Alabaster, AL). These were resuspended in chloroform and dried by nitrogen flow and vacuum overnight to a thin layer in glass tubes. Lipid stocks were sealed and left at −80°C for long-term storage.

### Peptide Expression and Purification

Isotopically single labeled ^15^N-VSTx1 was expressed and purified as previously described (Lau et al., 2016). A synthetic AA139 gene was introduced into both a pOPINE vector (Berrow et al., 2007) and a pLIC vector (Klint et al., 2013), containing a SUMO and MBP fusion tag, respectively. Initial expression tests show that soluble AA139 was only overexpressed when using the pOPINE expression vector with a SUMO tag. Subsequently we also screened the expression of VSTx1 in a similar N-terminal SUMO-tagged fusion expression vector (unpublished), which also produced high soluble protein yields (~0.5 mg per liter of culture). Recombinant ^15^N-AA139 was expressed as a His_6_-tagged SUMO fusion protein in *E. coli* (SHuffle T7 strain cells–NEB cat. C3029J) using standard biochemical methods. Details of the expression and purification methods are provided in the Supplementary Information.

### NMR Structure of AA139

1D ^1^H NMR experiments were recorded at different pHs and at pH 3.3 all non-exchangeable backbone amide protons could be observed. The spectra at pH 3.3 and 6.5 were superimposable indicating that the structure is unaffected by the change in pH. Subsequently, 2.5 mM synthetic AA139 (provided by Adenium) was analyzed in 20 mM phosphate buffer at pH 3.3 containing 5% D_2_O. All NMR experiments were performed on a Bruker Avance III spectrometer equipped with a cryogenically cooled triple resonance probe operating at a nominal ^1^H frequency of 700 or 900 MHz. The excitation sculpting sequence was used to suppress the solvent (H_2_O) resonance. Two-dimensional TOCSY [t_m_ (MLEV17 spin-lock mixing pulses) = 80 ms], NOESY [t_m_ (mixing time) = 300 ms], ^15^N-HSQC and ^13^C-HSQC were recorded at 25°C. Chemical shifts were directly (for ^1^H) or indirectly (for ^13^C, ^15^N) referenced relative to the 2, 2-dimethylsilapentane-5-sulfonic acid (DSS) signal at 0 ppm. The assignment of proton resonances was carried out using TOCSY and NOESY data using the CCPNMR software (Skinner et al., 2015). Torsion angles constraints were obtained using the TALOS+ software (Shen et al., 2009), and structure calculations were performed using CYANA 3.0 (Guntert, 2004).

#### Hydrogen-Deuterium Exchange Studies

All NMR spectra for the hydrogen-deuterium exchange studies were recorded on the spectrometer described above at 25°C. Spectra were referenced to DSS at 0 ppm. Lyophilized peptide was initially solubilized in 20 mM phosphate buffer at pH 3.3. The peptide was then lyophilized and subsequently dissolved in 100% D_2_O followed by immediate transfer to the spectrometer for measurement. 1D ^1^H and 2D TOCSY spectra were recorded at specific time points over a 24 h period. Hydrogen-deuterium exchange rates were measured by integrating each exchangeable amide resonance separately.

#### Amide Temperature Coefficient Studies

The temperature dependence of amide proton resonances was derived from 1D ^1^H to 2D TOCSY spectra recorded on a Bruker ARX 500 MHz spectrometer. Spectra were measured between 15° and 35°C, in 5°C increments, and referenced to DSS at 0 ppm. Assignment of the spectra was performed using the CCPNMR software (Skinner et al., 2015).

### NW9 Circularization

NW9 protein expression and purification followed standard procedures and is described in detail in the Supplementary Information, yielding 20 ± 5 mg of NW9 per liter of LB media. NW9 after TEV protease cleavage was buffer exchanged into the reaction buffer (20 mM Tris·HCl pH 7.5 and 150 mM NaCl) and then supplemented with 1 mM dodecyl-β-D-maltoside (DDM), 1 mM 2-mercaptoethanol and 10 mM CaCl_2_. 1:2 molar equivalents of evolved 5'-1' pentamutant Sortase A and NW9 were mixed and diluted to a total protein concentration of 15 μM. The reaction was left stirring for > 3 h at room temperature, for complete cyclisation of NW9 to cNW9. Subsequently, SM-2 Biobeads (Bio-rad) at 1 g/100 ml of reaction mixture were added to remove the detergent DDM from the reaction mixture. After incubation of 1 h of detergent absorption, the reaction mixture was filtered on a 0.45 μm polyethersulfone (PES) membrane and loaded into two 5 mL HisTrap Fast Flow Ni(II) columns (GE Healthcare) pre-equilibrated with reaction buffer to remove histidine tagged products including Sortase A, TEV protease and cleaved histidine tags. The flow-through containing cNW9 was collected and buffer exchanged to buffer A_ex_ (20 mM Tris·HCl pH 7.5, 1 mM DDM). The sample was purified and fractionated by anion-exchange chromatography using a HiScreen Q HP Column (GE Healthcare) using liquid chromatography (AKTAPurifier, GE Healthcare). After sample loading a linear gradient from 0 to 80% buffer B_ex_ (20 mM Tris·HCl pH 7.5, 500 mM NaCl, 1 mM DDM) was applied for 40 column-volumes.

### ND Production

To assemble NDs, dH5 or cNW9 and lipids were co-dissolved at [lipid]:[MSP] ratio of 50:1 in reconstitution buffer (20 mM Tris·HCl pH 7.4, 100 mM NaCl, 0.5 mM EDTA and 100 mM cholate) and mixed for 1 h at 4°C. A molar ratio of 1:50 (MSP:lipids) was calculated using the equation: N_L_×S = (0.423 × M-9.75)^2^, where N_L_ is the number of lipids per ND, M is the number of amino acids in the scaffold protein and S is the mean surface area per lipid used to form the lipid-nanodisc, measured in Å^2^ (Ritchie et al., 2009)–POPC and POPG have been estimated to have a similar mean surface area of around 70 Å^2^ (Janosi and Gorfe, 2010). Nevertheless, to confirm these calculations for the anionic lipid mixtures a range of MSP:lipid ratios (1:10, 1:30, 1:40, 1:50, and 1:80) were screened to monitor aggregation behavior. SEC chromatography of the assembled discs, showed high monodispersity in the 1:30–1:50 range, while 1:10 and 1:80 ratios produced chromatograms with evidence of high levels of inhomogeneity. Finally, the homogeneity of the lipid mixtures within the discs, was monitored by anion exchange chromatography and the elution profile was found to consist of a single peak, confirming efficient mixing of the lipids within the nanodiscs.

0.6 g of Bio-Beads SM-2 (Bio-rad) was added per mL of reaction volume, to absorb the detergent (cholate), and thus initiating ND assembly. The mixture was gently stirred for 4 h at 4°C for complete detergent removal. The solution was filtered through a 0.45 μm PES membrane to remove the Bio-Beads and then concentrated using centrifugal filtration (Amicon Centricon with a 10 kDa MW cut-off). The sample was buffer exchanged using a PD-10 column (GE Healthcare) into three different buffers: (i) 20 mM Tris·HCl pH 7.5, 50 mM NaCl, 1 mM EDTA; (ii) 20 mM NaPO_4_ pH 6.5, 50 mM NaCl, 1 mM EDTA; (iii) 20 mM Bis·Tris pH 6.5, 50 mM NaCl, 1 mM EDTA. After buffer exchange of NDs into one of three buffers, samples were concentrated by centrifugal filtration to ~5 mg/mL (unless otherwise stated) and stored at 4 °C.

### Electron Microscopy (EM)

Lipid NDs were diluted to a final concentration of 200 nM in 20 mM Tris–HCl, pH 7.5, 50 mM NaCl and adsorbed to glow-discharged and carbon-coated EM grids. Samples were prepared by conventional negative staining with 1 % (w/v) uranyl acetate. EM images were collected with a Tecnai 12 electron microscope operated at an acceleration voltage of 120 kV.

### Liquid Chromatography–Mass Spectrometry (LC-MS)

LC-MS analysis was conducted on lipid nanodiscs using Agilent Technologies 1200 Series Instrument with a G1316A variable wavelength detector set at λ = 210 nm, 1200 Series ELSD, 6110 quadrupole ESI-MS, using an Agilent Zorbax Eclipse XDB-Phenyl column (3 × 100 mm, 3.5 μm particle size, flow rate 1 mL/min, the mobile phases 0.05% formic acid in water and 0.05% formic acid in acetonitrile).

### Isothermal Titration Calorimetry (ITC)

The affinities of AA139 and VSTx1 for cNDs (both POPC and POPC:POPG mixtures) were determined using a Microcal iTC200 instrument (Malvern, UK). Experiments were performed in 20 mM Bis·Tris (pH 6.5), 50 mM NaCl and 1 mM EDTA. The peptides (at 350 μM) were titrated into 25 μM cNDs in 15 × 2.8 μl (AA139) and 19 × 2.2 μl (VSTx1) injections at 25 °C. Considering the symmetry of the cNDs the stoichiometry (*n*) was fixed at an even integer value (2, 4, 6 etc.). The concentration of the peptide was fixed and the effective concentration of the nanodisc allowed to vary together with the remaining variables. The effective nanodisc concentration was then fixed to the above determined value and all other parameters (including *n*) allowed to vary in order to determine the dissociation constants (*K*_d_) and enthalpy of binding (Δ*H*). During the fitting procedure the ITC response was integrated and normalized to a single-site binding model. The apparent binding free energy (Δ*G*) and entropy (Δ*S*) were calculated from the relationships Δ*G* = RTln(*K*_d_) and Δ*G* = Δ*H*-TΔ*S*. All experiments were performed at least in triplicate to ensure reproducibility of the data.

### NMR Titration

Solution NMR titration experiments between ^15^N-VSTx1 and ^15^N-AA139 and unlabelled cNDs [cNW9 (POPC/POPG (4:1))] were performed on a Bruker Avance III spectrometer equipped with a cryogenically cooled triple resonance probe operating at a nominal ^1^H frequency of 700 MHz. ^15^N-HSQC spectra were recorded at 25 °C. The concentration of ^15^N-VSTx1 or ^15^N-AA139 were kept constant at 20 and 40 μM respectively, while the concentration of cNDs [cNW9 (POPC/POPG (4:1))] was increased from 0 to 20 μM, with a total of up to six concentrations. A second titration of ^15^N-VSTx1 against cNDs [cNW9 (POPC)] was conducted under identical conditions to those noted above for this peptide. Each 2D experiment was acquired for ~ 45 min (16 scans and 75 complex points for AA139, and 32 scans and 37 complex points for VSTx1). All titrations were performed in 20 mM Bis·Tris buffer (pH 6.5), 50 mM NaCl and 1 mM EDTA.

NMR titration experiments between unlabelled VSTx1 or AA139 and ^15^N-cNDs [^15^N-cNW9 (POPC/POPG(4:1))] were performed on a Bruker Neo spectrometer equipped with a cryogenically cooled, triple resonance probe, operating at a nominal ^1^H frequency of 900 MHz, at 50°C. Three experiments were performed, the concentration of cNDs [^15^N-cNW9 (POPC/POPG (4:1))] was kept constant at 100 μM, while the concentration of VSTx1 or AA139 was increased from 0 to 50 then 100 μM. Each experiment was acquired over 4 h (128 scans and 50 increments).

### NMR Data Analysis

All spectra were processed using Topspin (Bruker Biospin) and the Rowland NMR toolkit (University of Connecticut). CCPNMR was used for spectral analysis. The change in peak intensity between titration points is the product of several dynamic processes that occur due to the binding event (for further details see Supplemental Discussion). The intensity of the signal is thus attenuated according to the weighted average of the free and bound states of the peptide:

(1) $$\begin{array}{r} I_{obs}=f_{F}I_{F}+f_{B}I_{B} \end{array}$$

The change in signal intensity due to this process *I*_*obs*_ can be related to the fraction of the peptide bound to the cND:

(2) $$\begin{array}{r} \Delta I_{obs}=f_{B}\Delta I_{\text{max}} \end{array}$$

*f*_*B*_ (fraction of the concentration of ligand bound to cNDs over the total ligand concentration- $\frac{\left[L_{B}\right]}{\left[L_{T}\right]}$) can then be fitted to a quadratic equation to obtain estimates of the dissociation constant, *K*_*d*_, and the number of equivalent binding sites on the cND, *n*, available for peptide binding (Granot, 1983; Williamson, 2013):

(3) $$f_{B}=\frac{(n\times [cND_{T}]+\left[L_{T}\right]+K_{d})-\sqrt{\left({\left(n\times [cND_{T}]+\left[L_{T}\right]+K_{d}\right)}^{2}-4\left[L_{T}\right]\times (n\times [cND_{T}]\right)}}{2\left[L_{T}\right]}$$

where [*L*_*T*_] is the total ligand concentration (^15^N labeled peptide), [*cND*_*T*_] is the total concentration of cND at each titration point, *I*_max_ is the normalized maximum intensity change and *I*_*obs*_ is the normalized change in signal intensity for a given spin pair and correlates with the mole fraction of peptide bound to the cND. It is assumed that (i) the signal observed is due to free ligand in addition to any residual signal from the bound ligand, due to fast local motion; (ii) at the highest concentration of nanodisc the binding is saturated (i.e., any signal remaining at highest cND conc. belongs to residues having fast local motion). Given the preceding conditions, normalization of the maximum-intensity-change ensures that the change in intensity observed is bounded between the free and saturated state (accounting for fast local motion). This allows us to fit data from all of the HSQC signals simultaneously. However, prior to this global fitting, each residue was fitted individually, those residues that yielded poor fits to the equation (i.e., produced very large fitting errors or negative *K*_*d*_ values) were excluded on the basis that additional processes were dominating the observed signal intensity change (other than chemical exchange due to binding). At this step residues with insufficient valid titration points (i.e., low initial intensity or very rapid decay, yielding fewer than three titration points above the noise level) and those from overlapping residues were removed. For all of the remaining residues a nearly constant value of *n* was found. The value of *n* was found to be close to an even integer, which agreed with the symmetry of the nanodisc, and the value of *n* was subsequently fixed to this nearest even integer value. Once this value was fixed a global fit of all of the data to equations 2/3 was performed. The fitting was performed using a Levenberg-Marquardt iterative non-linear least squares method (in Gnuplot v.3.5).

## Results

### cNW9 Production

**Figure 1:**
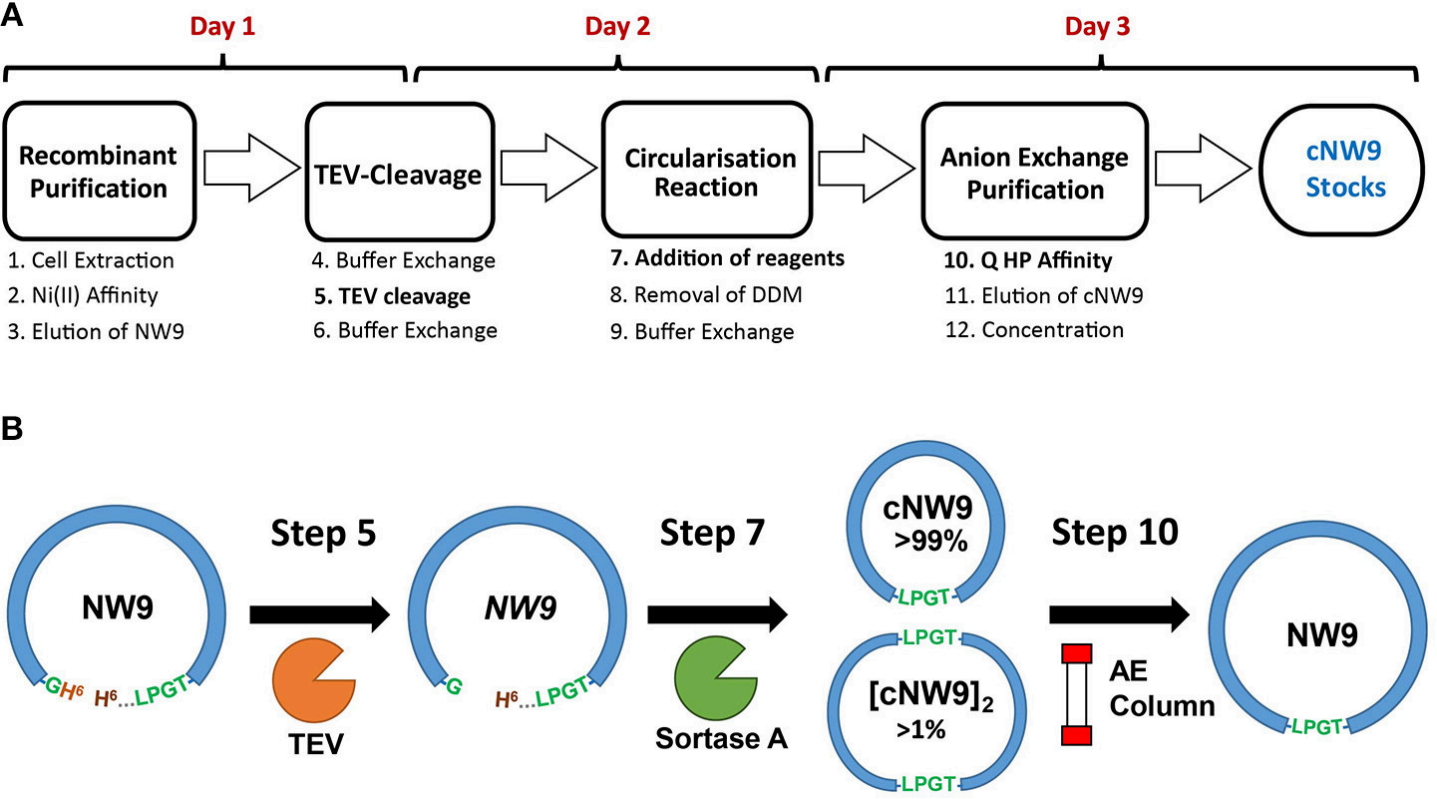
Production and circularization of cNW9. **(A)** Cell extraction of recombinant, noncircular cNW9 (NW9) followed by affinity purification (Ni Sepharose resin) and TEV cleavage (day 1 and overnight). TEV-cleaved NW9 circularization to a circular monomeric product (cNW9) and multimeric by-products ([cNW9]_n_) mediated by sortase A and assisted with lipid-detergents (day 2). Purification of cNW9 by anion exchange chromatography (Q HP resin) and concentration of protein stocks (day 3). **(B)** Reaction scheme for circularizing NW9 to cNW9 is illustrated with the key components/variants as designated in **(A)** (NW9, TEV, sortase A, anion-exchange column) and annotated with the key reaction steps in **(A)**.

MSP circularization following standard Sortase A reaction conditions (Tris·HCl, NaCl and CaCl_2_ conditions) (Nasr et al., 2017), led to a low yield of monomeric, cyclised, cNW9 (~3 mg/l of LB, corresponding to 15% of total NW9 produced). We observed that a large proportion of the reaction products were multimeric by-products (Mei and Atkinson, 2015; Henrich et al., 2017). To improve the yield of monomeric cNW9, we conducted a screen of reaction conditions including (i) temperature, (ii) total protein concentrations and (iii) different detergents/supplements added. Under optimized conditions, the reactions at 4 °C, room temperature and at 37°C were mostly complete in >24, ~3, and <1 h, respectively. Lowering the total protein concentrations from 30 to 5 μM led to detectable improvements in the fraction of monomeric products. A screening of detergents showed that non-ionic detergents such DM or DDM significantly improved the yield of monomeric cNW9 formed (from ~3 to ~6 mg/l of LB; or equivalently from 15 to 30% yield), while ionic detergents such as cholate had minimal effects on the final yield (see Figure S1A). Similar improvements in yields (doubling of the yield in presence of detergents) has been independently observed elsewhere using the non-ionic detergent Triton X-100 (Yusuf et al., 2018). In both cases the presence of detergents leads to near-complete removal of multimeric by-products. Following circularization, reverse nickel affinity and anion exchange chromatography, cNW9 is obtained at >99% purity (Figure 1 and Figure S1B). The assembly of the lipid cNDs routinely resulted in a yield of > 80% of pure lipid nanodiscs, regardless of the lipid composition.

### Nanodisc Stability Study

Three buffer conditions were chosen for the study of lipid-nanodiscs (ND) following the literature (Shenkarev et al., 2014; Susac et al., 2014) and NMR titration requirements:

- 20 mM Tris·HCl pH 7.5, 50 mM NaCl, 1 mM EDTA, a standard buffer-salt condition;
- 20 mM NaPO_4_ pH 6.5, 50 mM NaCl, 1 mM EDTA, a common NMR condition at a relatively low pH; and
- 20 mM Bis-Tris pH 6.5, 50 mM NaCl, 1 mM EDTA, where the low pH is retained while the favorable conductivity of the Tris based buffer is also retained.

Size-exclusion chromatography (SEC) was performed on ND samples at 5 mg/mL concentration, using a Superdex S200 5/150 *Increase* gel filtration column, pre-equilibrated in 20 mM Tris·HCl pH 7.5, 150 mM NaCl. Samples run over SEC were monitored and assessed for stability (all SEC traces can be found in Figures S2–S5).

To start, samples were stored at 4°C and monitored by SEC over intervals from day 1 to 30. The SEC profiles for circular cNW9 NDs were always sharper, indicating greater homogeneity of the sample. With the lipid composition consisting solely of POPC lipids, virtually no change in sample profiles was observed for cNW9 or dH5 NDs up to 30 days in the standard buffer condition (i).

cNDs were stable up to 30 days in all buffers tested. However, dH5 NDs in the lower pH conditions of (ii) and (iii) resulted in SEC profiles having an asymmetric peak, with a shoulder at shorter elution times (indicating either unassembled MSP or aggregates) after only 3 days of storage. It should be noted that unassembled MSP elutes earlier than the assembled NDs, indicating a larger effective molecular radius. Under these conditions the phosphate buffer condition (ii) seemed to be slightly worse for stability than the Bis-Tris buffer condition (iii).

The introduction of 20% POPG into the NDs did not affect the stability of cNW9 ND samples at 4°C. However, significant changes in the profile were observed in all buffer conditions for dH5 NDs at 4°C, with the observed effect getting worse from Tris (i) to Bis-Tris (iii) to phosphate (ii) buffer conditions. In the Bis-Tris buffer condition (iii), significant changes in the profile were observed for dH5 NDs at storage temperatures of 25° and 37°C. However, for cNW9 NDs, no changes were observed at either of these temperatures. Table 1 summarizes our observations, where the sample is deemed unstable if a clear change in the SEC profile can be seen, in particular noting the presence of shoulders or peaks at elution times earlier than that of the main ND peak.

**Table 1:** Summary of nanodisc stability under different storage conditions.

**Buffer**	**Temperature (°C)**	**Days stable/ total days**			
		**NDs with POPC**		**NDs with POPC:POPG (4:1)**	
		**dH5**	**cNW9**	**dH5**	**cNW9**
i. 20 mM Tris pH 7.5 *50 mM NaCl and 1 mM EDTA*	4	30/30	30/30	1/30	30/30
ii. 20 mM PO_4_ pH6.5 *50 mM NaCl and 1 mM EDTA*	4	3/30	30/30	1/30	30/30
iii. 20 mM Bis-Tris pH6.5 *50 mM NaCl and 1 mM EDTA*	4	3/30	30/30	1/30	30/30
	25	–	–	1/2	2/2
	37	–	–	0/2	2/2
	−20	–	–	2/2	2/2

*Circular and linear (cNW9 and dH5) nanodiscs containing either zwitterionic (POPC) or anionic [POPC:POPG (4:1)] lipid mixtures were stored at different temperatures in different buffers (all including 50 mM NaCl and 1 mM EDTA). The stability of the discs was monitored by size-exclusion chromatography using an S200 Increase 5/150 column (GE Healthcare), at daily intervals*.

**Figure 2:**
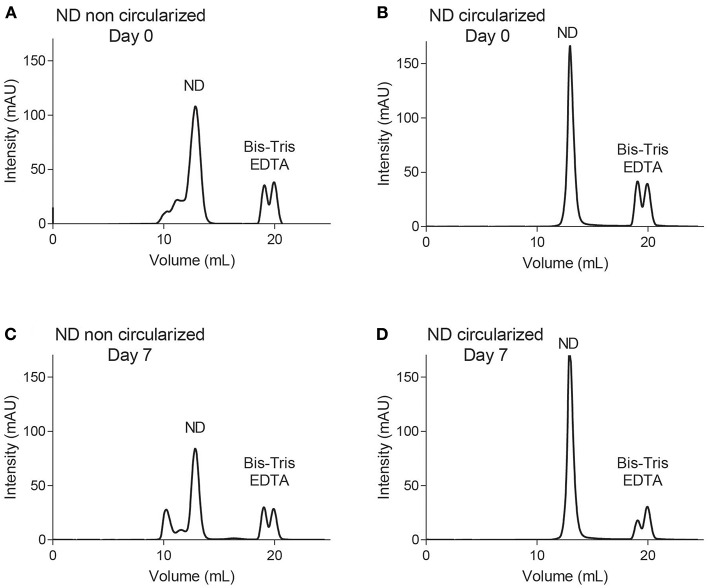
Size exclusion chromatography using an S200 Increase 10/300 column. Traces of NDs containing POPC/POPG (4:1) lipids using membrane scaffold proteins dH5 **(A,C)** and cNW9 **(B,D)** at day 0 **(A,B)** and day 7 **(C,D)** after storage at room temperature.

A detailed study was conducted for both constructs of NDs containing POPC/POPG (4:1) in 20 mM Bis-Tris pH 6.5, 50 mM NaCl, 1 mM EDTA using an analytical Supderdex S200 10/300 *Increase* column. Note that this column provides a higher resolution than the smaller S200 5/150 column used in our screening experiments described above (i.e., traces in Figures S2–S5). At assembly time (day 0), both constructs were analyzed by SEC, using the 10/300 column pre-equilibrated in 20 mM Tris-HCl pH 7.5, 150 mM NaCl (Figures 2A,B). The NDs were left at room temperature for seven days and reanalyzed via SEC (Figures 2C,D). Each fraction was subjected to LC-MS (Figures S6–S7). Negative stain EM was also performed for selected fractions of NDs [dH5 (POPC/POPG (4:1))], confirming the presence of NDs of increased diameter after 7 days of storage (Figure S8). The dH5 ND samples aggregated over time, while no degradation was observed for cNW9 NDs.

### Recombinant Peptide Production and Solution Structure Determination

VSTx1 was produced using an MBP-fusion construct as previously described, where the structure of this peptide is also reported (Lau et al., 2016)–yielding ~1 mg of peptide per liter of bacterial culture. VSTx1 was also produced using a SUMO-fusion construct. The smaller fusion partner further simplified the purification step by HPLC, where MBP would often be present as a significant and difficult to remove contaminant. The SUMO fusion of VSTx1 yielded ~0.5 mg of pure peptide per liter of culture.

AA139 was expressed in *E. coli* SHuffle cells transformed with a pOPINE-His_6_-SUMO-AA139 plasmid vector. The transformed cells were cultivated in minimal medium containing ^15^N-NH_4_Cl. The His_6_-SUMO-AA139 fusion protein was obtained in the soluble fraction after cell lysis. Following Ni^2+^ affinity chromatography and cleavage with SUMO protease, AA139 was purified via reversed-phase HPLC. The final yield was approximately 1 mg of ^15^N-AA139 per liter of culture. The purity and mass were analyzed by LC-MS. The mass of the peptide is 2548.1 g/mol. The experimentally measured m/z values of ^15^N-AA139 (647.9 [+4], 518.6 [+5] and 432.3 [+6]), match the calculated values (647.8, 518.4 and 432.2). The purity of both peptides was >99% (as measured by HPLC).

Chemical shifts of the protons of AA139 were measured from 2D ^1^H–^1^H TOCSY and NOESY NMR spectra. ^13^C and ^15^N shifts were obtained from 2D HSQC spectra (Table S1 – BMRB ID: 30260). Secondary Hα shifts (Figure S9) show that AA139 is composed of two β-strands and a turn forming a β-hairpin structure (Wishart et al., 1995), further supported by hydrogen bonds (see Tables S2, S3), The chemical shifts were used to predict backbone dihedral angles (TALOS), and were subsequently used in structure calculations. The peptide structure is further stabilized by two disulfide bridges Cys^3^-Cys^20^ and Cys^7^-Cys^16^, these were defined by three upper and three lower distance restraints between the heteroatoms, and included in the structure calculations. The above restraints were supplemented with NOE based distance restraints and the NMR solution structure of AA139 (PDB 5V11), was calculated by torsion angle dynamics (CYANA–see also Figure S10 and Table S4). The two β-strands cover residues Cys^3^-Arg^10^ and Arg^13^-Cys^20^. The β-strands are intervened by a type I' β-turn (Asn^11^/Gly^12^) forming slightly twisted anti-parallel β-strands in the β-hairpin structure. The distortion created by the right-handed twist of the two-stranded β-sheet results in an amphipathic structure, commonly observed in membrane interacting peptides.

### Peptide-Lipid Interactions by ITC and NMR

cNDs containing a zwitterionic (POPC) or an anionic lipid mixture [POPC:POPG (4:1)] in the above described Tris-Bis buffer (20 mM BisTris (pH 6.5), 50 mM NaCl and 1 mM EDTA–used in all subsequent experiments) were used to study the interactions between the cNDs and the membrane-active peptides (VSTx1 or AA139) by isothermal titration calorimetry (ITC) and NMR. Neither peptide showed binding to zwitterionic cNDs by ITC.

Initial NMR experiments were conducted to find concentration ranges where clear intensity changes could be observed (data not shown). Based on these concentrations, a titration was conducted where the concentrations of uniformly isotopically labeled ^15^N-VSTx1 or ^15^N-AA139 was fixed at 20 and 40 μM, respectively, and NMR spectra were acquired in the presence of increasing concentrations of cNDs.

#### ITC Results

The ITC binding isotherms using anionic cNDs produced results consistent with weak binding in the μM range (Figure S11). During the fitting, first the stoichiometry for the binding of VSTx1 to cNDs was determined to be 2 peptides per disc [i.e., other even integer values used produced larger errors, see also Materials and Methods section Isothermal Titration Calorimetry (ITC)]. Using this information, the effective concentration of cNDs in the ITC experiment was determined (4.54 μM). This is roughly 1/5 of the concentration determined by measurement of absorbance at 280 nm from the MSPs, and consistent with the POPG ratio within the discs. The experiment was repeated with different starting concentrations of peptides and nanodiscs, in all cases yielding the same fitted parameters (data not shown). The effective cND concentration determined by the fitting procedure was used for all subsequent modeling, where, instead the stoichiometry was allowed to vary. This yielded a stoichiometry of 4 AA139 peptides to each cND. The ITC data are shown in Figure S11 and the fitting of these data to the binding isotherm summarized in Table 2 (see also Table S5).

**Table 2:** Thermodynamic parameters for VSTx1 and AA139 binding to cNDs.

**Peptide**	***K*_**d**_**	**ΔH**	**–TΔS**	**ΔG**	***n***
	***μM***	***kcal/mol***	***kcal/mol***	***kcal/mol***	
VSTx1	2.02 ± 0.81	−1.92 ± 0.73	−5.88 ± 0.94	−7.80 ± 0.22	2.00 ± 0.20
AA139	1.16 ± 0.28	−6.29 ± 0.61	−1.83 ± 0.75	−8.12 ± 0.16	4.09 ± 0.09

*The average and standard deviation of three replicates are presented. Fitted values of the dissociation constant (K_d_), enthalpy (ΔH) of binding and stoichiometry (n), as well as the calculated binding free energy (ΔG) and entropy (-TΔS) are reported*.

#### ^15^N HSQC Titrations of VSTx1

**Figure 3:**
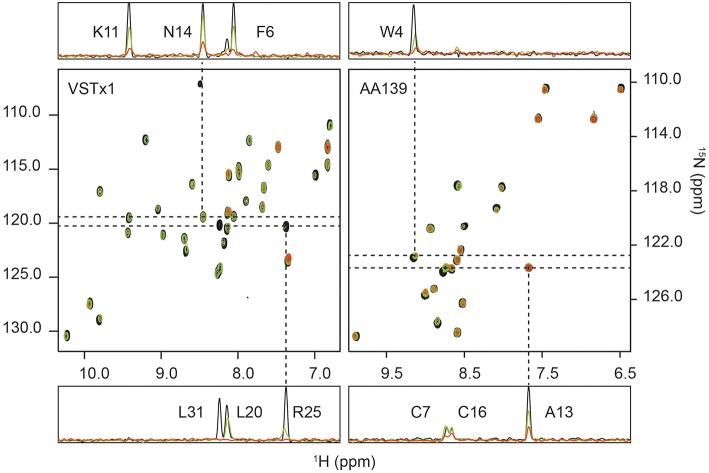
^1^H-^15^N HSQC titrations of membrane-active peptides against nanodiscs containing bacterial model membranes. (Left) The titration of ion-channel inhibitor VSTx1 (20 μM) against increasing concentration of nanodiscs [POPC:POPG (4:1)]. The three superimposed spectra correspond to a [peptide]:[nanodisc] ratio of 1:0 (black), 1:0.25 (green) and 1:1 (red). (Right) The titration of antimicrobial peptide AA139 (40 μM) against increasing concentration of nanodiscs [POPC:POPG (4:1)]. The three superimposed spectra correspond to a [peptide]:[nanodisc] ratio of 1:0 (black), 1:0.25 (green) and 1:0.375 (red). In both cases 1D traces are shown at ^15^N frequencies corresponding to peaks that show a small change in intensity that does not fit to the binding isotherm (N14 for VSTx1 and Ala13 for AA139), and a signal (Arg25 for VSTx1 and Trp4 for AA139) having a strong intensity change that fits the binding isotherm. The latter also display an observable chemical shift and linewidth change consistent with intermediate exchange due to binding. Assignments are provided in 1D traces.

^15^N-VSTx1 at a concentration of 20 μM was titrated in a series against anionic cNDs [POPC:POPG (4:1)] with increasing concentration: 0, 5, 10, and 20 μM (20 mM BisTris (pH 6.5), 50 mM NaCl and 1 mM EDTA). Each of the four samples was then used to acquire a 2D ^1^H-^15^N-HSQC experiment. A concentration dependent broadening of the signals was observed (see Figure 3). There were also minor chemical shift changes in a concentration dependent manner for signals that experienced significant line-broadening. The fitting was performed as described (see Materials and methods), where the backbone amide of Leu31 was excluded due to a very sharp signal loss leading to insufficient points for fitting (only 2 points above the noise–taken as 3 times the noise level). Glu2, Lys5, Ser13, and Ser24 were completely undetectable under conditions tested. Resonances of Cys10 and Asn12 were removed due to overlap.

The initial fit of individual residues to Equations (2) and (3) showed that residues Cys3, Asn14, Asp15, Cys16, and Phe35 as well as the sidechain resonance of Asn14 did not produce reliable fits to the model. The remaining residues fit the binding isotherm having, *n* = 2.3±0.7 (equivalent binding sites). The resonances of Gly4, Met7, Trp8, Ser23, Arg25, Trp26 and the sidechain of Trp28 all decayed rapidly with increased cND concentration, and these only had three valid titration points (missing a signal above noise level at the highest concentration of nanodisc). The value of, *n*, was subsequently fixed to 2 (even integer) for a global fit of all remaining resonances containing four valid data points after normalization, i.e., Phe6, Lys9, Lys11, Cys17, Lys18, Asp19, Leu20, V21, Cys22, Lys27, Trp28, Cys29, Val30, Ala32, Ser33, and sidechain NH resonances of Trp8, Asn12 and Trp26. This yielded an equilibrium dissociation constant, *K*_d_, of 1.11 ± 0.13 μM–using a constant value of 2 equivalent binding sites (Figure S12).

Further to the above titration, a second series of experiments were conducted with cNDs containing zwitterionic lipids. It had previously been observed that VSTx1: 1) binds to zwitterionic micelles, and 2) binds to the MSP of NDs (Shenkarev et al., 2014; Lau et al., 2016). The titrations were conducted under identical conditions as those described above for the anionic lipid NDs. This approach allows us to isolate the effect of the anionic lipids from any binding to zwitterionic lipids and MSP as well as potentially providing insights into the binding of the peptide to neutral lipids. The results show a significantly weaker binding than that observed for the anionic lipids (note that this binding was undetectable using ITC). The average change in peak height between the control experiments and the lowest concentration of added nanodisc (5 μM) is 38% (standard deviation of 15%) for the anionic lipid mixture and only 5% (standard deviation of 10%) for the zwitterionic mixture (in both cases removing L31 as an outlier). Only a few peaks displayed significant intensity changes (>10%) in the zwitterionic lipid mixture at this concentration of cNDs (backbone amides of Gly4, Phe6, Lys9, Lys11, Arg25, Trp26, Leu31, Ala32 as well as the sidechain amides of Trp8 and Trp26). Interestingly, the pattern of peak intensity changes remains similar in both cases, indicating that the binding mode is conserved. We also note that the majority of these amino acids could be fitted to the binding isotherm in the previous titration.

#### ^15^N HSQC Titrations of AA139

^15^N-AA139 (40 μM) was titrated in the presence of cNDs [POPC:POPG (4:1)] at 0, 5, 7.5, 10, 12.5, 15 and 20 μM. For each sample a 2D ^1^H-^15^N HSQC spectrum was acquired. The increasing concentration of the ND added did not result in any significant chemical shift changes, however, significant broadening of the signals was observed in a concentration dependent manner (see Figure 3). The change in intensity was modeled using a quadratic equation to obtain estimates of the stoichiometry and dissociation constant of the interaction (Equations 2, 3). As noted in the methods section, several residues were removed for either yielding too few data points (backbone amides of Phe2, Asn11 and Gly12) or due to overlap (Val6 and Cys7). The initial fitting led to the exclusion of residues that did not fit to the model (Cys3, Ala8, Arg9, Arg10, Ala13, Arg14, Cys16, and Cys20). The line-broadening in these residues cannot be modeled by equations (2) and (3), either due to an insufficient change in intensity across the concentration range tested or the presence of other exchange processes. The remaining seven residues all fit the model well and produced values of equivalent binding sites, *n*, of 3.9 with a standard deviation of 0.3. This value was then fixed to 4, and after normalization of the observed maximum height change, a global fit of the data for all residues was performed, yielding an equilibrium dissociation constant, *K*_d_, of 0.41 ± 0.13 μM (Figure S12).

**Figure 4:**
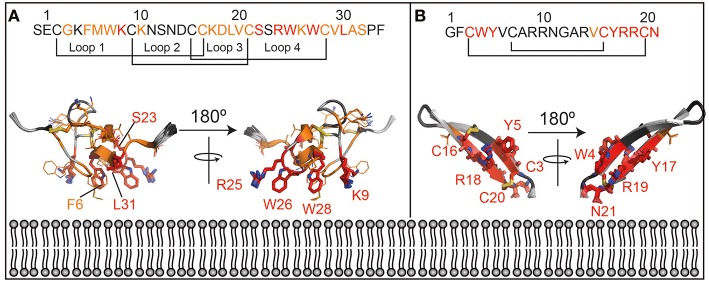
Lipid interaction of membrane-active peptides. Cartoon representation of the peptides [**A** = VSTx1 (PDBID: 2N1N), **B** = AA139 (PDID: 5V11)] are oriented to show the likely binding orientation with respect to the lipid bilayer (bottom) based on the NMR titration data (180° rotation also shown). Disulfide bonds are shown as sticks. The peptide sequences and numbering are shown above the peptides. The residues in red (sidechains as sticks and labeled) are those that show the strongest perturbation upon addition of nanodiscs, those in orange show an intensity change consistent with the binding isotherm (sidechains as sticks) and residues in dark gray show smaller perturbation that do not fit the binding isotherm. Residues that were not included in the analysis (missing/overlapped) are shown in light gray. Structures in panels **(A)** and **(B)** are not to the same scale.

Further, a qualitative analysis of the likely binding interface of the peptide was performed by comparing the intensity of the signals at the first two titration points (in the absence of cNDs and in the presence of 5 μM of cND). Residues that are most strongly affected at this low concentration of ND, are most likely to be at the binding interface. These residues include the hydrophobic and acidic residues at the termini of the β-hairpin loop (see also Figure 4).

### Peptide-MSP Interactions by NMR

As noted above it had previously been suggested that there is an interaction between the anionic MSP and cationic peptides such as VSTx1 and AA139 (Shenkarev et al., 2014). A titration series of unlabeled peptides at 0, 50 and 100 μM against cNDs [^15^N-cNW9: (POPC)] at 100 μM was conducted in the Tris-Bis buffer (iii) described above. The titration was followed by 2D ^1^H-^15^N TROSY experiments acquired at 50 °C for 4 h. The spectra featured well dispersed peaks for ^15^N-cNDs at this temperature (at 25 °C the signals were unresolvable). In both cases, there were no peptide-concentration dependent chemical shift or intensity changes, suggesting that there is little or no interaction between the MSP and the peptides (Figure S13). The sample stability was monitored before and after each experiment by SEC and 1D ^1^H NMR (not shown), and the NDs were found to be intact in all cases (see also Figure S14).

## Discussion

### Production and Stability of Anionic NDs

The procedure described here for production of cNDs, departs from the original protocol described (Nasr et al., 2017). Here, a sortase inhibitor was not required and lipid-detergents (1 mM DDM) were used in the cyclisation reaction, which not only allows for higher protein concentrations in the circularization reaction but also leads to improved yields of the monomeric product, likely due to disruption of the formation of dimeric MSPs in solution. Our results are consistent with the independently developed protocol by Yusuf et al. where introduction of Triton X-100 (1 mM) was shown to significantly reduce multimeric by-products when trying to cyclise small MSP constructs through the same sortase-mediated reaction (Yusuf et al., 2018). The convergence of these methods on the optimal concentration of detergents during cyclisation is an encouraging sign of arrival at a general approach to generating high yields of cNDs.

In this work, we investigated the stability of NDs containing anionic lipid mixtures commonly used to model the bacterial membrane [POPC:POPG (4:1)], and compare this to NDs containing zwitterionic lipids (POPC). The stability of the NDs is evaluated based on EM images and SEC profiles, where in the latter aggregation or disassembly results in asymmetric elution profiles with increased absorbance at early elution times. For the linear NDs there is a general trend that the lower pH buffers result in higher heterogeneity of the NDs (Figures S2–S3). The level of heterogeneity increases over time and after ~ 3 days we find that in the lower pH buffers (Bis-tris and phosphate at pH 6.5) the shoulders of the ND elution peaks increase in intensity sufficiently to be observed as distinct peaks. These trends are exacerbated in the presence of anionic lipid mixtures (in linear NDs), where at low pH there is clear evidence of aggregation/disassembly even at day 0 (Figure S3), in all buffers tested (result of all observations summarized in Table 1 based on data in Figures S2–S5). We note that the observed heterogeneity would render most biophysical measurements unfeasible.

Fortunately, the introduction of head-to-tail cyclisation of the MSP resulted in excellent (c)ND stability regardless of the lipid composition or sample conditions–i.e., no changes were observed to the SEC elution profiles or EM images. Our conclusion based on these results is that small linear NDs are unsuitable for studies of anionic lipid bilayers. We further note that, although it is known that the presence of anionic lipids does not alter the membrane thickness or its fluidity, it has a very significant effect on the stability of lipid nanodiscs in solution. This conclusion may shed some light into previous work on the use of anionic NDs for studies of membrane-active peptides, where it was found that VSTx1 binds very effectively to anionic NDs, and based on analysis of NMR titration data a stoichiometry of ~35 peptides to each ND was found (Shenkarev et al., 2014). This value appears physically unlikely considering that the peptide diameter can be estimated to be ~20 Å (measured from the pdb structure 2N1N across the orange region in Figure 4A) and the ND used in the reported study has an available lipid diameter of ~ 58 or 35 Å when excluding one or two shells of MSP bound lipids, respectively (Hagn et al., 2018). Based on our findings (although using a smaller ND) the apparently high stoichiometry may have been a consequence of aggregation due to instability of the linear NDs used. Indeed, despite the inherent experimental limitations at the observed binding affinities, our NMR and ITC data are both consistent with two independent binding sites for VSTx1 on cNW9 (available lipid diameter of cNW9 estimated to be 43 or 26 Å when excluding one or two shells of MSP bound lipids, respectively; Hagn et al., 2018), which provides a physically more realistic binding mode.

Although protein interactions with NDs have been studied previously by ITC (Agamasu et al., 2017), to our knowledge, ITC has not previously been utilized to quantify interactions between membrane-active peptides and NDs, and our results demonstrate that it may prove to be a valuable tool in future studies of such peptides. Finally, we also acquired dynamic light scattering (DLS) data for our samples to determine if this could be used to distinguish assembled and unassembled NDs. However, for these small NDs, the size difference between the MSP and the ND was too small to generate a reliable difference in scattering (data not shown).

### Expression and Binding of Membrane-Active Peptides to Bacterial Model Membranes

Isotope labeling of both membrane-active peptides was achieved through recombinant protein expression in bacteria. In the case of AA139, a SUMO-fusion tag produced high soluble yields (~1 mg per liter of culture) when expressed in SHuffle cells, in contrast to the MBP fusion expressed in the BL21 strain, where no soluble protein was found. It is worth noting the inherent challenge in finding a suitable bacterial expression system for an antimicrobial peptide. In this case it appears that the SUMO-fusion may have reduced the toxicity of the peptide. Although the bacterial expression of VSTx1 had previously been described, we also found that VSTx1 could be produced in relatively high yields using this strategy (SUMO-fusion in SHuffle cells–yielding 0.5 mg per liter of culture).

Transferring the results of the analysis of the NMR data (see discussion in Supplementary Information), onto the structure of the two peptides, allows us to gain some insights in the likely binding pose of the two molecules (Figure 4). In particular, those residues that show large intensity changes and fit the binding isotherm [fall into category (c or d) in Supplemental Discussion] are most likely to be in the binding interface and are shown in red with their sidechains displayed as sticks. These residues also show the highest intensity changes at the lowest concentration of NDs added.

Those that fit the binding isotherm but show smaller changes in their intensity [category (a) in Supplemental Discussion] are likely to experience membrane binding to some extent, and these are shown in orange with sidechains as lines. Those that show relatively small intensity changes, and do not fit the binding isotherm [category (b) in Supplemental Discussion] are least likely to be close to the binding interface and are shown in dark gray without sidechains indicated. Residues that were not included in the analysis are shown in light gray. This data is consistent with the interpretation that the red residues are inserting into the membrane while the orange residues are at the lipid/water interface.

The lipid binding data for VSTx1 are consistent with previous findings, and show, perhaps unsurprisingly, that the positively charged peptide binds more strongly to lipids containing negative head-groups. What is surprising, however, is that the same amino acids show the strongest perturbations in both titrations (against anionic and zwitterionic bilayers). This would suggest that the binding is driven by the hydrophobic patch of the peptide (loops 1 and 4) and that the affinity is enhanced by the basic residues in these loops in the presence of acidic moieties at the bilayer surface. This is particularly interesting as this peptide has been found to interact with acidic residues on its ion-channel receptor, in what appears to be a conserved mode-of-action in the inhibitory function of gating-modifier toxins (Lau et al., 2016; Zhang et al., 2018). It is also worth noting that the peptide had previously been found to bind zwitterionic micelles with moderate affinity by NMR spectroscopy (Ozawa et al., 2015; Lau et al., 2016). In contrast, we find that the peptide binds very weakly to zwitterionic lipids in cNDs and that this binding is undetectable by ITC. These results are more consistent with the centrifugation assays performed using liposomes (where no partitioning into zwitterionic liposomes is observed). Taken together, this suggests that the peptide has a stronger affinity for micelles than for bilayers–it remains to be seen if this is a general finding for membrane-active peptides or a property of VSTx1. Interestingly, however, the micelle binding interface identified by NMR (Ozawa et al., 2015; Lau et al., 2016) is the same as the cND interface identified here. Thus, although in this case, we find that the peptide has a stronger affinity for micelles than lipid bilayers, it appears that the binding mode is conserved.

The structure of AA139 reveals a twisted β-hairpin structure similar to other members of this family of antimicrobial peptides (Edwards et al., 2016). In contrast to the related arenicin-2 peptide, however, we do not observe any evidence of the peptide disrupting lipid membranes (from EM data–Figure S14). The NMR data suggest that the termini of the peptide are the likely interface with the lipid bilayer. This segment of the protein contains a hydrophobic N-terminus and a basic C-terminus, which are both likely to contribute to its interaction with the anionic lipid bilayer. An interesting observation is that the negatively charged C-terminus is likely to form unfavorable interaction with both the head-groups and the tails of the lipids, and that amidation of this residue may enhance its affinity toward bacterial membranes. The binding pose also suggests that the long axis of the peptide is likely to be closer to being perpendicular to the plane of the bilayer, than in the plane. This may provide some support for the higher stoichiometry (of peptide to disc) derived for this peptide from the NMR data when compared to VSTx1.

The ITC experiments revealed that both peptides had binding affinities in the low μM range. Interestingly, although the binding free energy (ΔG) is similar in both cases (~-8 kcal/mol), the relative contribution of entropic and enthalpic terms is different. The binding of VSTx1 appears to be largely driven by an entropic component (~-6 kcal/mol) rather than an enthalpic component (~-2 kcal/mol). The larger entropic term is consistent with increased disorder either in the peptide or the lipid bilayer upon binding. Although it is difficult to deduce the relative contributions of each of these components, we do see a very significant broadening of residue L31 of VSTx1 even in the presence of small concentrations of cNDs. This is consistent with a conformational change upon binding, and is likely to account for some of the observed gain in entropy. The change in conformation of a leucine residue is likely to occur in a highly apolar environment and the data would suggest that this face of the molecule partitions into the lipid bilayer.

In contrast to VSTx1, the ITC results for AA139 reveal a reversal of the relative thermodynamic terms, with a greater enthalpic term (~-6 kcal/mol). AA139 has a net charge of +5 which is due to the presence of five arginine residues accounting for almost a quarter of the total amino acid composition. This would suggest that the binding of the peptide is driven by charge-charge interactions at the lipid interface, involving some of these residues. As noted above the masking of the C-terminal charge through amidation would increase the net charge to +6 which may further strengthen the enthalpic contribution to the binding energy.

In general, there is good qualitative agreement between the ITC and the NMR data. The binding constants derived from the NMR data were as expected exaggerated when compared to the ITC data, thus, although not quantitative provide a reasonable estimate of the binding. The stoichiometry was consistent when comparing data from the two methods. We note that in both cases the relatively weak binding observed, required careful analysis of the data, where a priori knowledge regarding the symmetry of the disc was used to improve the fitting of the data. Thus, although there is good agreement in the values derived, we note that these data are near the limits of binding interactions that can be detected by the two methods and some uncertainties are likely associated with the inherent sensitivity of these techniques in this binding regime.

## Conclusion

We present a method to obtain high yields of cNDs in 3 days and assess their suitability for biophysical studies in solution. We performed size-exclusion chromatography (SEC) of linear and cyclised NDs of different compositions under varying conditions. The cNDs consistently displayed significantly improved stability over their noncircular counterparts. The study revealed that linear NDs can be unstable under common experimental conditions, particularly in phosphate buffers at a low pH and when containing anionic lipids. In contrast, cNDs were stable at all solution conditions and lipid compositions tested. In addition, the sharper peaks observed in the SEC profiles indicate greater homogeneity. Finally, we describe a method for high-yield (~mg per liter of culture) recombinant production of two membrane-active peptides, notably including an antimicrobial peptide–AA139.

These materials are then used to evaluate the use of biophysical methods to study the membrane binding properties of membrane-active peptides against cNDs containing anionic lipid mixtures, that approximate the charge distribution of bacterial membranes. We use ITC to measure the binding thermodynamics, and heteronuclear 2D NMR to characterized the binding of ^15^N-labeled peptides against cNDs. We first study the well characterized VSTx1 peptide to validate the proposed approach, and find good agreement with previous reports while revealing new information regarding the thermodynamics of the binding event. We then apply our method to gain insights into the activity of AA139, an antimicrobial peptide with an unknown mode-of-action. ITC and NMR data show that AA139 binds to the cNDs with low μM affinity, which is driven by a significant enthalpic contribution (−6 kcal/mol) and a stoichiometry of 4 peptides per disc. We further solved the structure of AA139 by NMR, which revealed a twisted β-hairpin fold, and allowed us to determine the likely lipid-binding interface of the peptide, which included the tails of the antiparallel β-sheets. These results establish the use of cNDs in combination with ITC and solution-state NMR as a novel and general method for investigating the membrane binding properties of membrane-active peptides.

## Author Contributions

AZ, IE, and MM conceived the study. AZ, IE, BM, GS, MH, and XJ performed the experiments. AZ, IE, BM, MH, BC, and MM analyzed the data. IE, AZ, and MM wrote the paper with input from all authors. All authors contributed to different components of the study design.

### Conflict of Interest Statement

The authors declare that the research was conducted in the absence of any commercial or financial relationships that could be construed as a potential conflict of interest.

## Supplementary Material

The supporting information include further details on the NMR analysis, ITC data, size exclusion chromatography profiles, thin layer chromatography images, EM negative staining images and LC-MS traces.

## Abbreviations

Nanodisc: ND
circular MSP1D1ΔH5: cNW9
linear MSP1D1ΔH5: dH5
antimicrobial peptides: AMPs
membrane scaffold protein: MSP
protein databank: PDB
dodecylphosphocholine: DPC
1,2-dimyristoyl-sn-glycero-3-phosphocholine, dodecyl-β-D-maltoside (DDM), decyl-β-D-maltoside (DM): DMPC
1,2-Dimyristoyl-sn-glycero-3-phosphorylglycerol: DMPG
palmitoyloleoyl-phosphatidylcholine: POPC
palmitoyloleoyl-phosphatidylglycerol: POPG
size exclusion chromatography: SEC
electron microscope: EM
mass spectrometry: MS
chemical shift perturbation: CSP
polymerase chain reaction: PCR.
