# Supplementary material for: Elucidating the Lipid Binding Properties of Membrane-Active Peptides Using Cyclised Nanodiscs
Source: Front Chem. 2019 Apr 16;7:238. doi: 10.3389/fchem.2019.00238 (PMC6477933; doi:10.3389/fchem.2019.00238)
Supplement: Supplementary file 1 [file Data_Sheet_1.PDF]

Elucidating the lipid binding properties of membrane active peptides using cyclised nanodiscs

Alan H. Zhang[†1], Ingrid A. Edwards[†2], Biswa P. Mishra[1], Gagan Sharma[1], Michael Healy[2], Alysha G. Elliot, Mark A. T. Blaskovich[2], Matthew A. Cooper[2], Brett Collins[2], Xinying Jia[1] and Mehdi Mobli[1]*

[†] *These authors contributed equally to this work*

1. Centre for Advanced Imaging and 2. Institute for Molecular Bioscience

The University of Queensland, St Lucia, 4072, QLD

**m.mobli@uq.edu.au*

Supplementary information

Supplemental methods

AA139 expression and purification

E. coli SHuffle competent cells (NEB C3029J) transformed with pOPINE-His$_6$-SUMO-AA139 were grown in 10 mL of LB media with 100 µg/mL of ampicillin at 37 °C with shaking at 220 rpm until OD_{600} reached late exponential phase (OD_{600} = 0.8). 3 mL of the culture was then transferred to 50 mL of "minimal media" (MM) (Marley et al., 2001) with 100 µg/mL of ampicillin supplemented with 1 g/L $^{15}NH_4Cl$ and grown at 37°C with shaking at 250 rpm overnight. The 53 mL culture was then transferred into 2 L of MM with 100 µg/mL of ampicillin and grown at 37°C with shaking at 250 rpm until reaching an OD_{600} of 0.8. The culture was then cooled down to 20°C, followed by induction by addition of 0.5 mM isopropyl β-D-1-thiogalactopyranoside (IPTG) at 20°C with shaking at 250 rpm overnight. The cells were harvested by centrifugation at 5,000 *g* for 30 min at 4°C and resuspended in lysis buffer (150 mM sodium phosphate (pH 8), 300 mM NaCl) supplemented with 20 mM imidazole and EDTA-free protease inhibitor cocktail (Sigma-Aldrich). The cells were then disrupted using a cell disruptor at 27 KPsi for 1 cycle and subjected to centrifugation at 30,000 *g* for 30 min at 4°C. The supernatant was applied to two 5 mL Histrap Fast Flow Ni columns (GE Healthcare) (pre-equilibrated with lysis buffer) on an AKTAPurifier FPLC instrument (GE Healthcare). His-tagged recombinant protein was eluted with lysis buffer supplemented with 500 mM imidazole and the fractions corresponding to the fusion protein were collected and concentrated by centrifugal filtration using an Amicon Centricon (Merck) filter with a 3 kDa molecular weight (MW) cut-off. Buffer exchange was performed by dialysis using a 6-8 kDa MW cut-off membrane for 6 h against 2 L of the cleavage buffer of 50 mM Tris-HCl (pH 8), 150 mM NaCl. SUMO protease was then used for the cleavage of the fusion protein with a (w/w) ratio of 1:50, shaking overnight at 4°C. The solution was loaded onto a Gilson HPLC system using C18 column (Zorbax® C18, 9.4 x 100 mm, 5 µm) equilibrated with solution A (H_2O containing 0.1% trifluoroacetic acid). The chromatography was performed at a flow rate of 3 mL/min with a linear gradient of solution B (100% acetonitrile containing 0.1% trifluoroacetic acid): 5% for 6 min, 5–50% for 16 min, 50–100% for 4 min, 100% for 2 min and 5% for 3 min. The peaks monitored at 210 nm were collected and lyophilized. The lyophilized product was then analyzed by LC-MS ^1H and ^1H-^{15}N-HSQC NMR spectra were acquired for comparison to the synthetic equivalent.

NW9 protein purification

E. coli BL21(DE3) competent cells transformed with pET28a-NW9 were grown in LB media with 50 µg/mL of kanamycin at 37 °C with shaking at 220 rpm until reaching an OD_{600} of 0.8. Induction was innitiated by the addition of 1 mM IPTG at 37°C with shaking at 250 rpm for 3 h. The cells were harvested by centrifugation at 5,000 *g* for 30 min at 4°C and resuspended in lysis buffer (50 mM Tris pH 8.0, 500 mM NaCl) supplemented with, 1 mg/mL lysozyme, 1% Triton X-100, 1 tablet of EDTA-free protease inhibitor cocktail (10 mL of lysis buffer per 1 g of cell pellet) and for 30 mins at 4°C. Cells were lysed by sonication on ice (40% power, 1 s on and 3 s off for a total of 15 min). The sonicated sample was centrifuged at 40,000 *g* for 30 min at 4°C. The supernatant was filtered by a 0.45 µm polyethersulfone (PES) membrane and was loaded into 2 x 5 mL HisTrap Fast Flow Ni columns (pre-equilibrated with lysis buffer) at 3 mL/min. The columns were washed with multiple rounds (5 CV each) of wash buffers sequentially in the order: a. lysis buffer supplemented with 1% Triton X-100; b. lysis buffer supplemented with 50 mM Sodium Cholate; c. lysis buffer; d. lysis buffer supplemented with 20 mM Imidazole. Histidine tagged NW9 was eluted with elution buffer (lysis buffer supplemented with 500 mM imidazole). Fractions that were

>95% pure, as judged by sodium dodecyl sulfate polyacrylamide gel electrophoresis (SDS-PAGE) analysis, were consolidated, supplemented with 50 mM EDTA and then buffer exchanged over an Amicon Centricon with a 10 kDa filter with cleavage buffer (25 mM Tris.HCl pH 7.5, 150 mM NaCl). The sample was then incubated with 1:50 molar ratio of TEV protease to NW9 and 2 mM dithiothreitol (DTT). Cleavage was conducted under dialysis (6,000 MW dialysis bag) against 2 L of cleavage buffer overnight. The cleaved NW9 sample was confirmed by SDS-PAGE analysis and spun down at 3,000 *g* for 5 min and 4°C to remove minor precipitates.

Supplemental discussion:

Interpretation of NMR titration data

The rate of exchange due to binding ($K_{binding}$) in relation to the difference in chemical shifts ($\Delta\delta$) between the free and bound state, determines the behaviour of the NMR signals in a titration experiment. In both of our titrations, we observed loss of signal intensity and line-width broadening with increasing stoichiometry of nanodiscs. This is typically observed when $K_{binding}$ is similar to $\Delta\delta$, and suggests binding in the μM range (intermediate exchange). The titration data further revealed a non-uniform (across different resonances), but concentration dependent loss of signal intensity suggesting additional contributions leading to enhancement of the relaxation properties of the different nuclei. Thus, the signal-intensity-loss will be due to one or more of the below occurring due to binding:

(i) a chemical-exchange process (in the μs-ms timescale),
(ii) the change in effective correlation time upon binding,
(iii) changes in the local chemical environment leading to changes in the dipolar couplings to nearby nuclei;
(iv) a conformational-exchange process (in the μs-ms timescale);

From the above we would expect all residues to experience processes (i) and (ii) to some extent depending on their proximity to the binding interface and their local dynamics, and for most residues this would be a dominant contribution to the relaxation enhancement. The chemical-exchange process may be observed by measurements of changes in the linewidth, but the extent of this varies due to the presence of the other processes above making accurate measurements impractical. Instead, we would expect that the change in intensity for those residues where the relaxation enhancement is dominated by processes (i) and (ii)/(iii) can be fitted to the binding model (eq. 2 and 3) – although these may be overestimated due to compounding effects of (ii) and (iii) on the chemical exchange process being modelled (i), resulting in a lower K_d. Residues at the binding interface (iii) will likely also show chemical shift changes depending on the exchange rate (if sufficiently large these will also fit the binding isotherm and are used preferentially where fast exchange is observed). Residues where the relaxation enhancement is significantly affected by (iv) are, however, not likely to produce reliable fits to the model (these will be a mixture of two exchange processes). Due to these complexities, model fitting is not a reliable method for extracting accurate K_d values from NMR titrations in the intermediate exchange regime – either resulting in overestimation of affinities or yielding a poor fit to the binding isotherm (Williamson, 2013), and it other measurements such as ITC are preferred. However, we note that the NMR data acquired contains valuable information pertaining to the binding event, including stoichiometry, and with the above constraints considered, we have performed a qualitative analysis of our binding

data. Our analysis distinguishes between the following scenarios, where the observation is given in standard font and the interpretation given in italics:

(a) Residues that show relatively small changes in intensity and can be fitted to the model. *Residues near but not at the binding interface [(i) and (ii)/(iii)].*
(b) Residues that show relatively small changes in intensity and cannot be fitted to the model. *Residues far from the binding interface with high local motion [(ii) only].*
(c) Residues that show relatively large changes in intensity and can be reliably fitted to the model. *Residues that are likely to be in the binding interface [(i), (ii) and (iii)].*
(d) Residues that show relatively large changes in intensity but cannot be fitted to the model. *Residues that may be undergoing a conformational change with a different rate to the above processes [(i), (ii), (iii) and (iv)].*

From the above, those in categories (c) are most likely to be in the binding interface. For AA139 the tails belong to category (c), whereas the loop most likely belongs to category (b). For VSTx1, residues in the N/C termini and loop 2 are most likely in category (b), those in loop 4 show the largest intensity changes and most likely fit into category (c), and those in loops 1 and 3 best fit category (a). There is some evidence that some residues may fall into category (d), and these include Ala8 and Arg18 in AA139 and Phe35 for VSTx1, that all show relatively large intensity changes but do not fit the binding isotherm.

Supplamental references:

Marley, J., Lu, M., and Bracken, C. (2001). A method for efficient isotopic labeling of recombinant proteins. *J Biomol NMR* 20**,** 71-75.

Williamson, M.P. (2013). Using chemical shift perturbation to characterise ligand binding. *Progress in Nuclear Magnetic Resonance Spectroscopy* 73**,** 1-16.

Supplemental Figures:

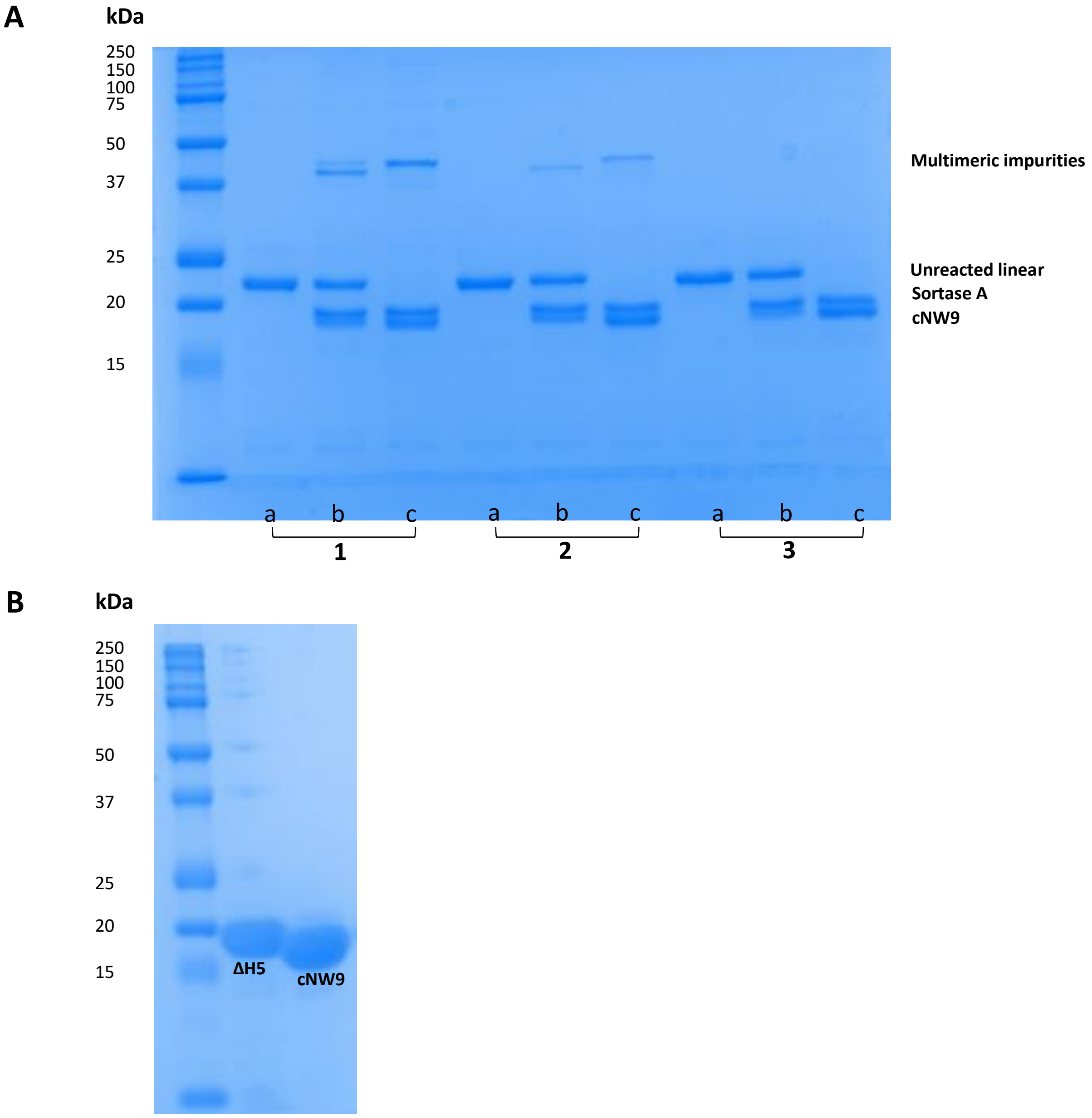

Figure S1 – SDS-PAGE gels. (A) NW9 circularisation conditions: 1. buffer (20 mM Tris.HCl pH 7.5, 100 mM NaCl, 15 mM $CaCl_2$); 2. buffer and 1 mM cholate; 3. buffer and 1 mM dodecyl-mocyel detergent. Each condition has fractions for (a) before reaction, (b) 1 h after reaction and (c) 12 h after reaction. (B) Comparison of MSP1D1ΔH5 and cNW9 protein stocks used in this study after purification. Ladder marker provided on the left (Precision Plus Protein^{TM} Prestained Standards, Bio-Rad).

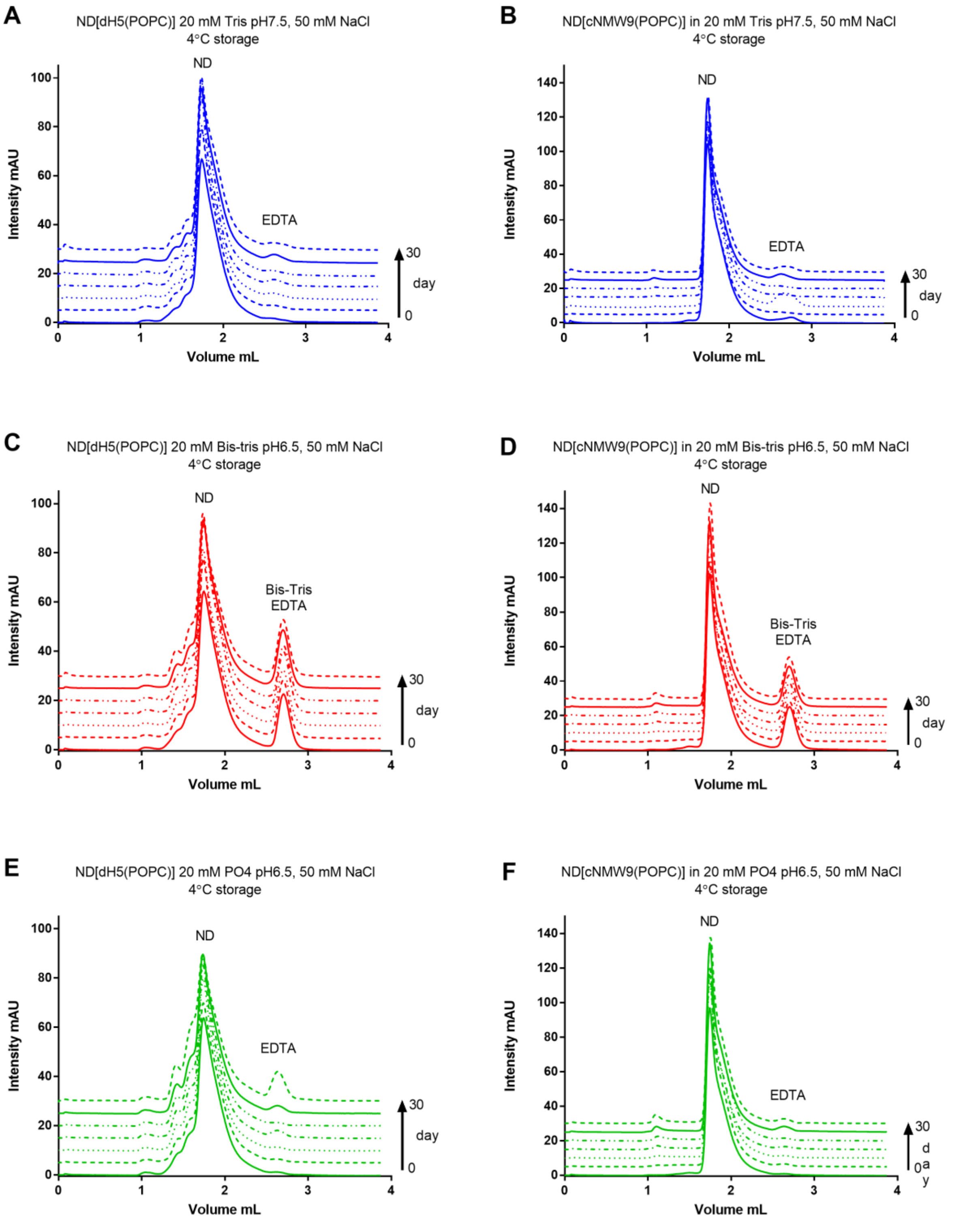

Figure S2 – Nanodiscs, composed of POPC, stability over 30 days at 4°C storage. (A), (C) and (E) use dH5 construct while (B), (D) and (F) use cNW9 construct. From top to bottom; (A) and (B) Tris buffer pH7.5 (blue), (C) and (D) Bis-Tris buffer pH 6.5 (red), (E) and (F) PO4 buffer pH 6.5 (green).

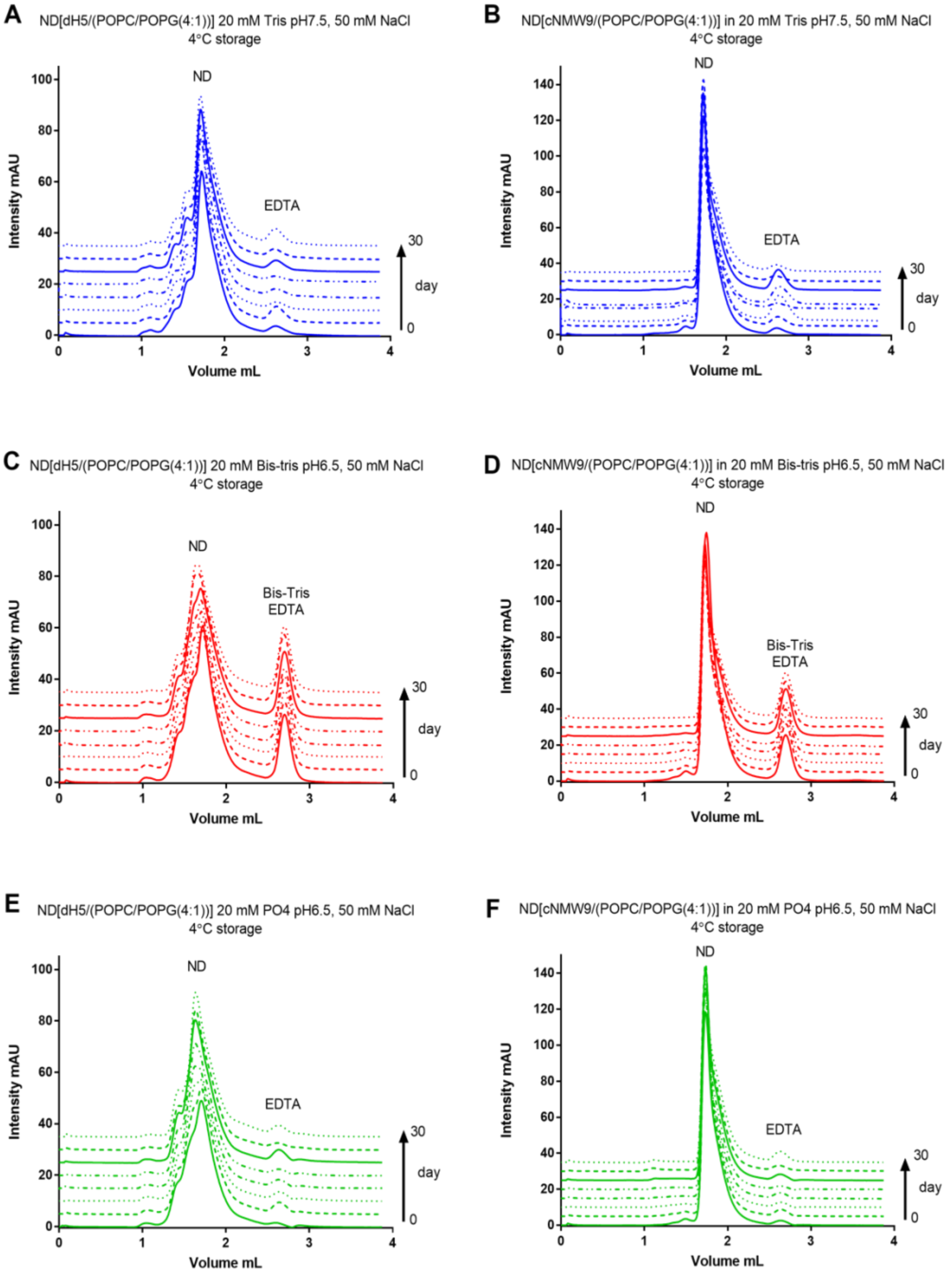

Figure S3 – Nanodiscs, composed of POPC/POPG (4:1), stability over 30 days at 4°C storage, using a 10/300 Increase column. (A), (C) and (E) use dH5 construct while (B), (D) and (F) use cNW9 construct. From top to bottom; (A) and (B) Tris buffer pH7.5 (blue), (C) and (D) Bis-Tris buffer pH 6.5 (red), (E) and (F) PO4 buffer pH6.5 (green).

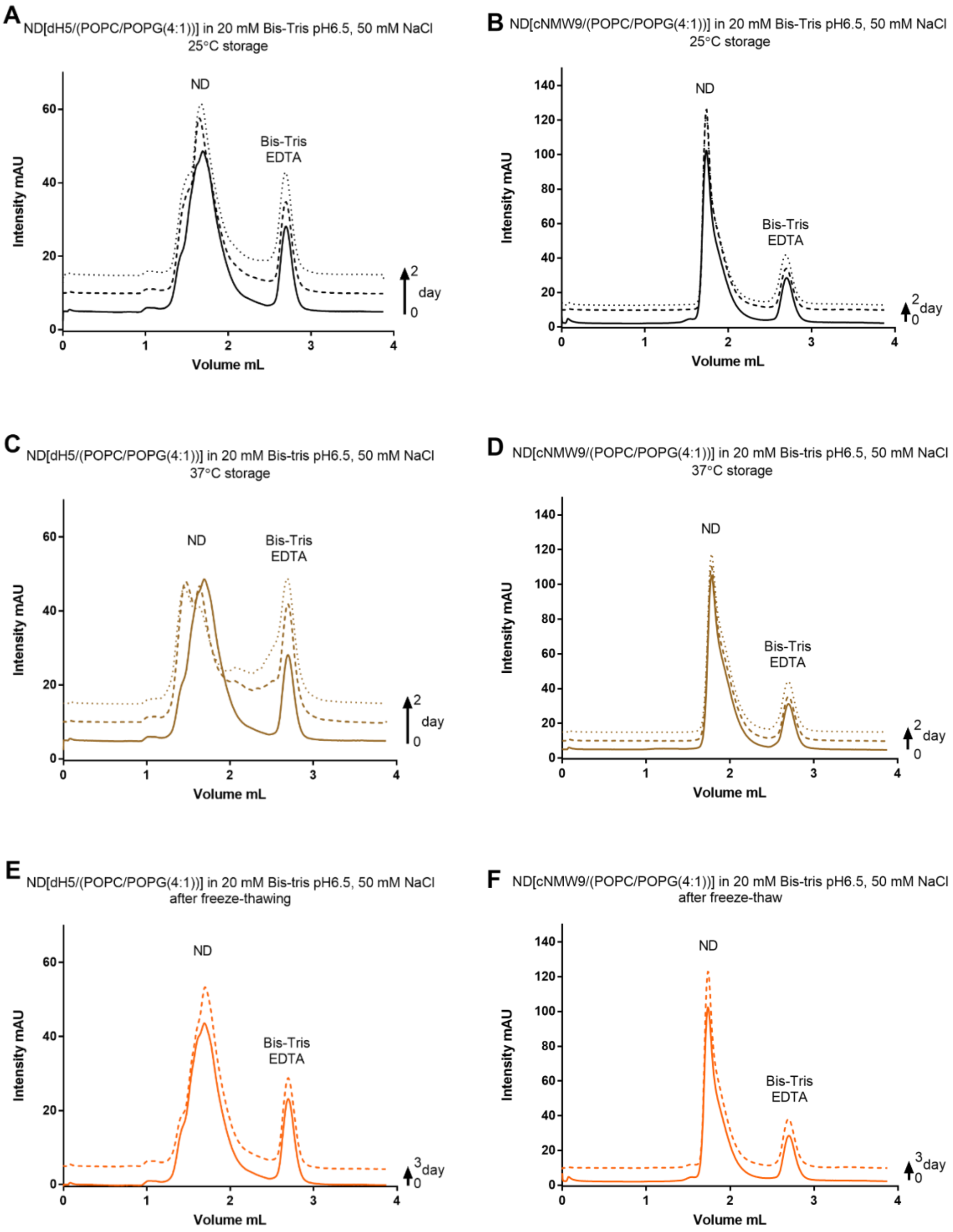

Figure S4 – Nanodiscs, composed of POPC/POPG (4:1) in Bis-Tris buffer pH 6.5, stability over 2-3 days, using a 10/300 Increase column. (A) and (B) 25°C, (C) and (D) 37°C, (E) and (F) after freeze-thawing. (A), (C) and (E) use dH5 construct while (B), (D) and (F) use cNW9 construct.

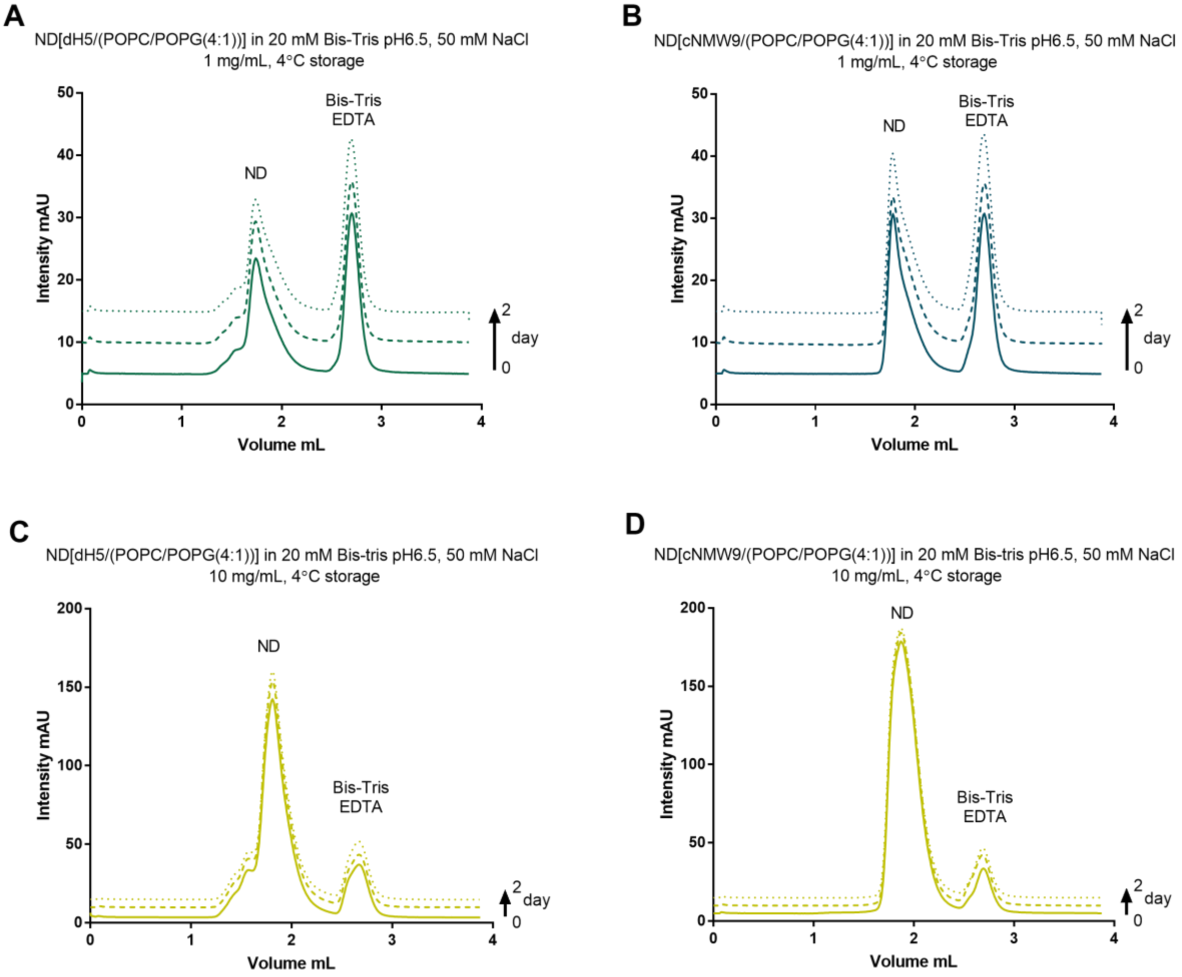

Figure S5 – Nanodiscs, composed of POPC/POPG (4:1) in Bis-Tris buffer pH 6.5, stability over 2 days, using a 10/300 Increase column. (A) and (B) stored at 1 mg/mL, (C) and (D) stored at 10 mg/mL. (A) and (C) use dH5 construct while (B) and (D) use cNW9 construct.

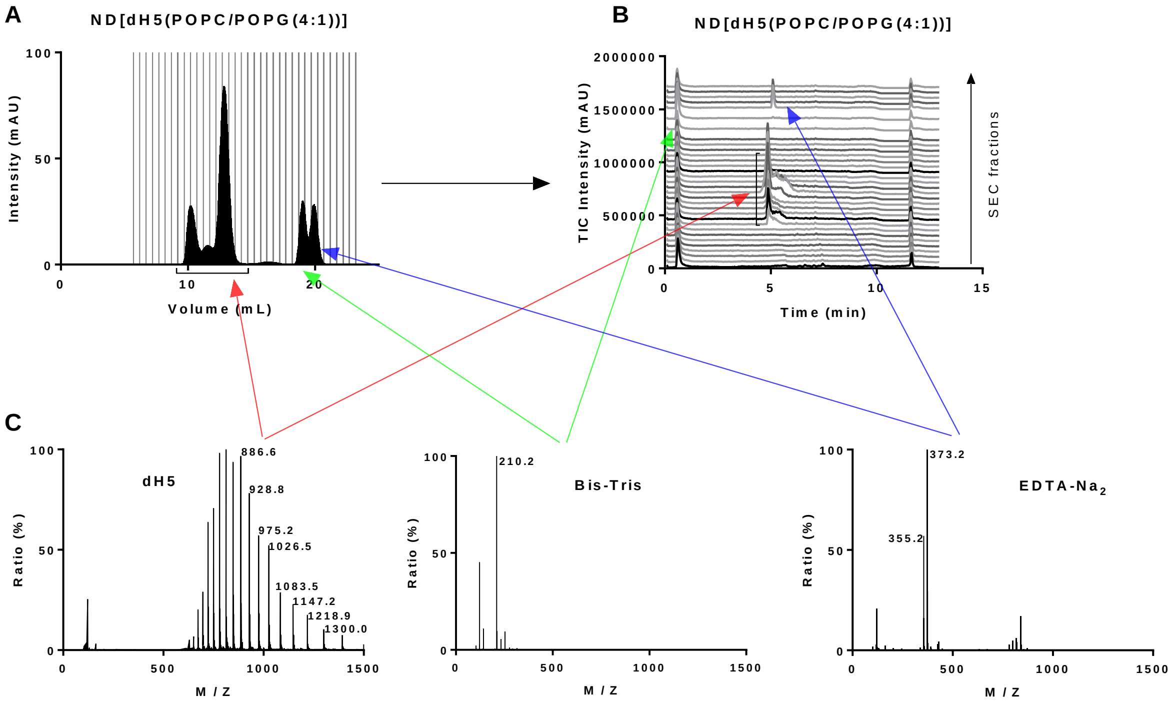

Figure S6 – (A) SEC of ND[dH5(POPC/POPG(4:1))] nanodiscs at day 7 stored at room temperature, (B) vertical stacking of LC-MS (ESI in positive mode) of the SEC fractions and (C) M/Z TIC trace of selected peaks. MW(dH5) = 19486 g/mol, MW(Bis-Tris) = 209.2 g/mol and MW(EDTA-Na_2) = 372.2 g/mol.

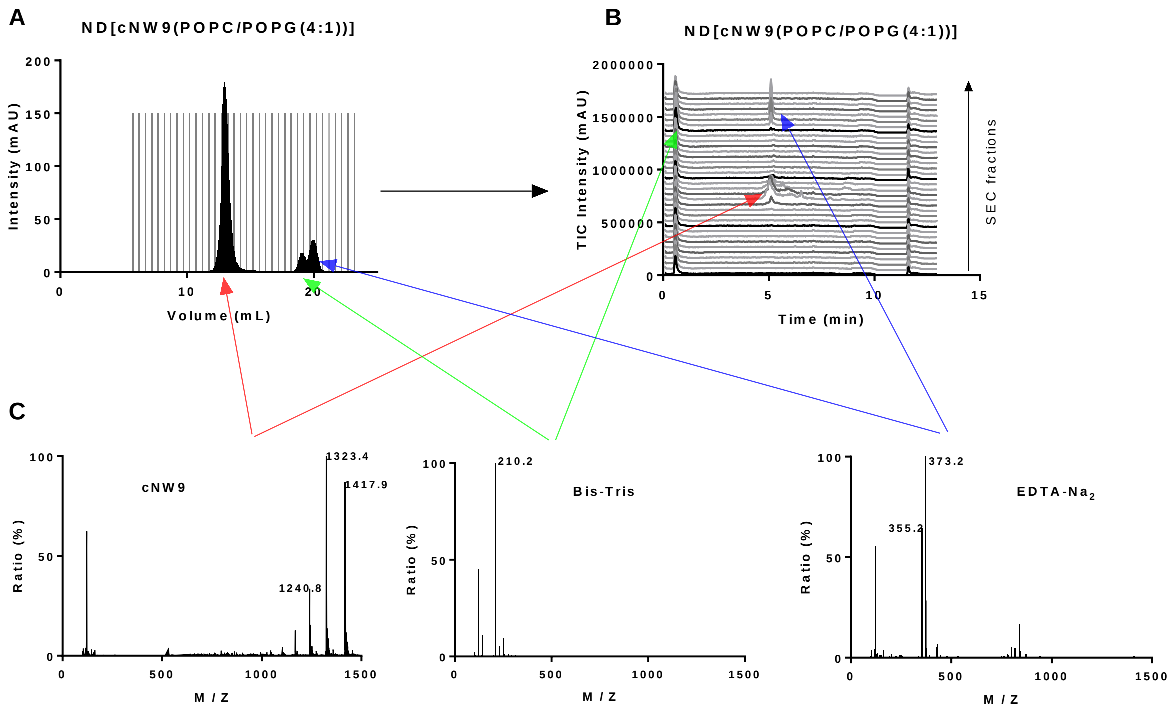

Figure S7 – (A) SEC of ND[cNW9(POPC/POPG(4:1))] nanodiscs at day 7 stored at room temperature, (B) vertical stacking of LC-MS (ESI in positive mode) of the SEC fractions and (C) M/Z TIC trace of selected peaks. MW (cNW9) = 19837 g/mol, MW (Bis-Tris) = 209.2 g/mol and MW (EDTA-Na_2) = 372.2 g/mol.

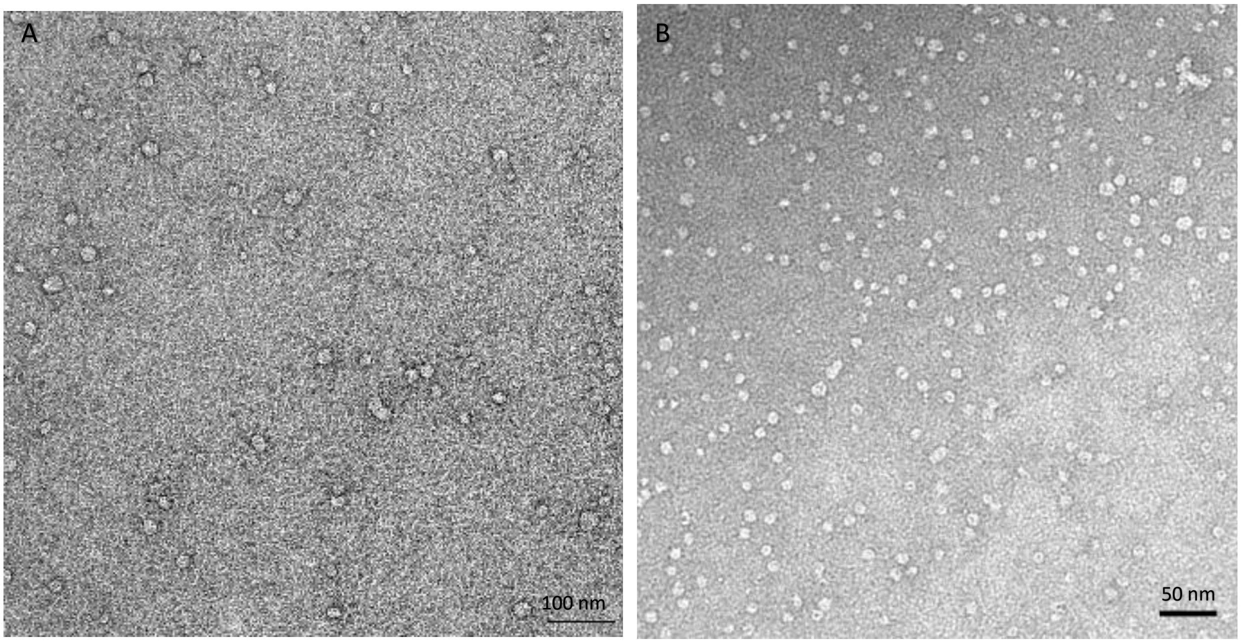

Figure S8 – Negative staining electron microscopy of SEC of ND[dH5(POPC/POPG(4:1))] nanodiscs at day 7 stored at room temperature Figure S8. (A) fraction 10 showing ND aggregates and (B) fraction 16 showing homogeneous ND, of ND[dH5(POPC/POPG(4:1))] at day 7 after storage at room temperature, Figure 5-10(A).

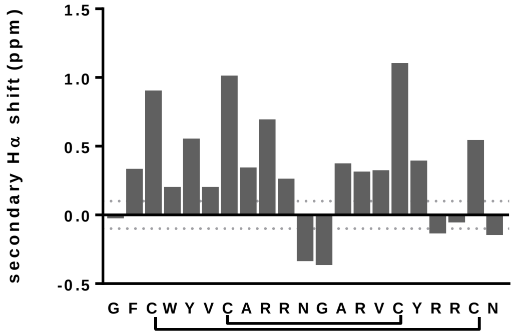

Figure S9 – Secondary Hα shifts (variation from random coil) for AA139.

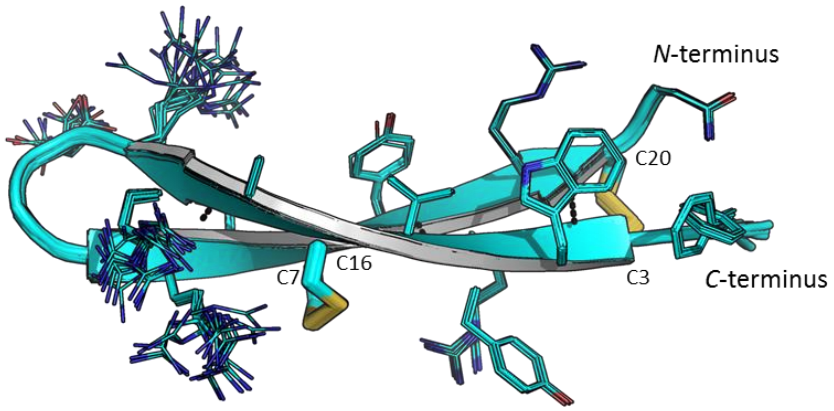

Figure S10 – Ensemble of 20 3-dimensional NMR solution structures of AA139. Structures (PDB: 5V11) are displayed in cartoon form showing the classic ß-hairpin assembly created by anti-parallel ß-sheets and two stabilizing disulfide bonds (yellow) across the sheets, key residues labelled for orientation and the *N*- and *C*-terminus. Images created using PyMol.

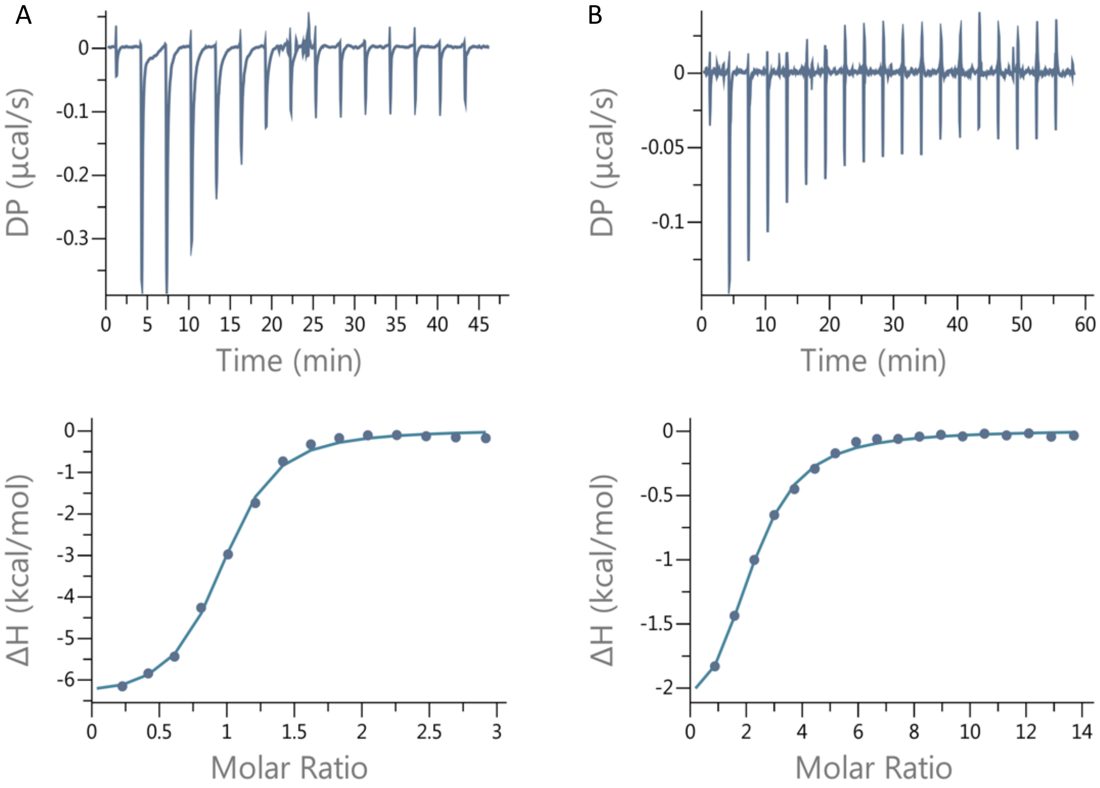

Figure S11: ITC measured interactions between peptides and cNDs. A) A representative biding curve of AA139 binding to nanodiscs measured at 25°C. B) A representative binding curve of VSTx1 binding to nanodiscs measured at 25°C.

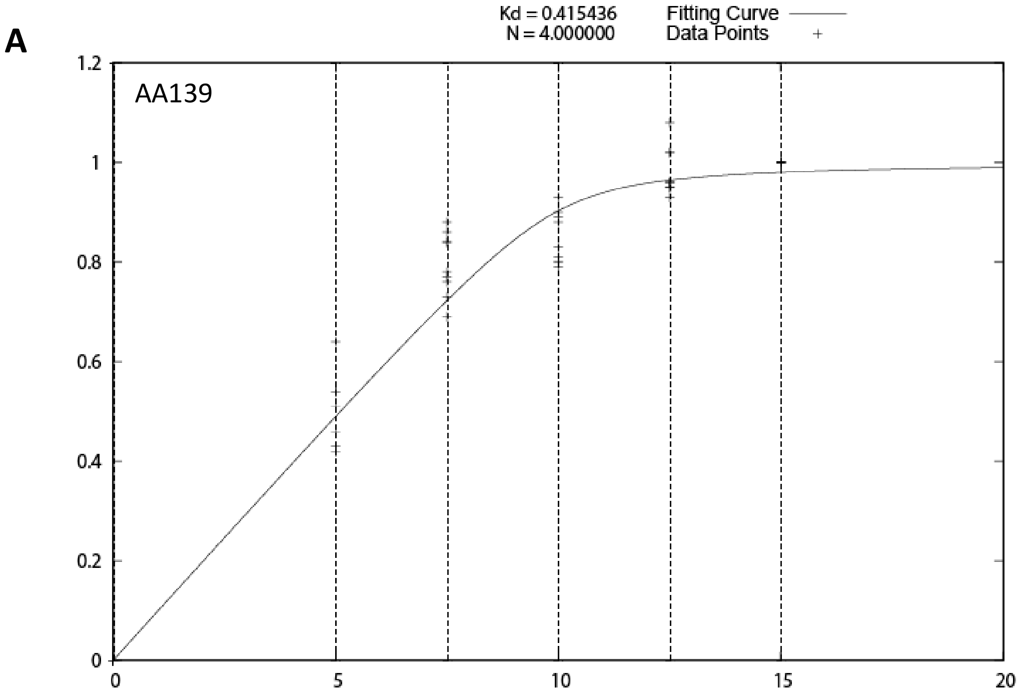

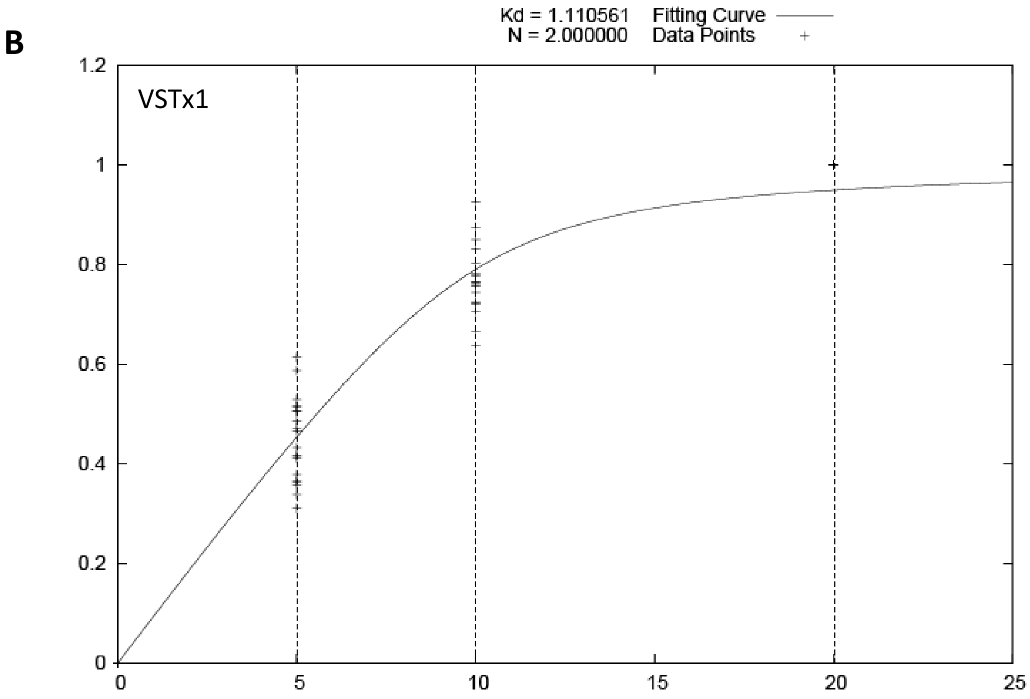

Figure S12 – Binding constant fitting for the NMR titration experiment between peptide and ND[cNW9(POPC/POPG(4:1))] for (A) AA139 and (B) VSTx1.

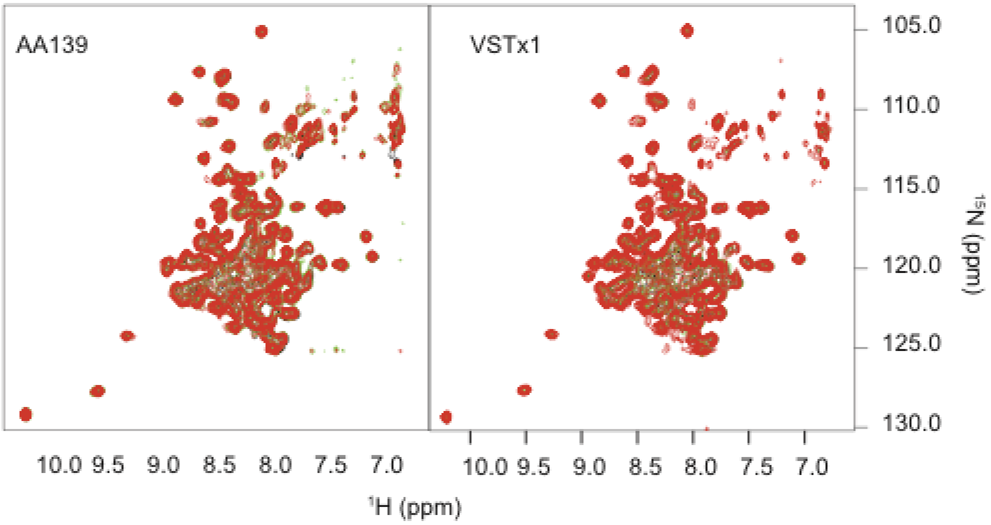

Figure S13 – ^{15}N-TROSY-HSQC of ^{15}N labelled cNW9 in nanodiscs containing zwitterionic (POPC) lipids. The concentration of the labelled protein is kept constant at 100 µM while increasing concentration of AA139 or VSTx1 is added (black: 0 µM of peptide, green: 50 µM of peptide, red: 100 µM of peptide). There was no peptide-concentration dependent change in chemical shifts or peak intensities. The spectra were recorded at 50°C.

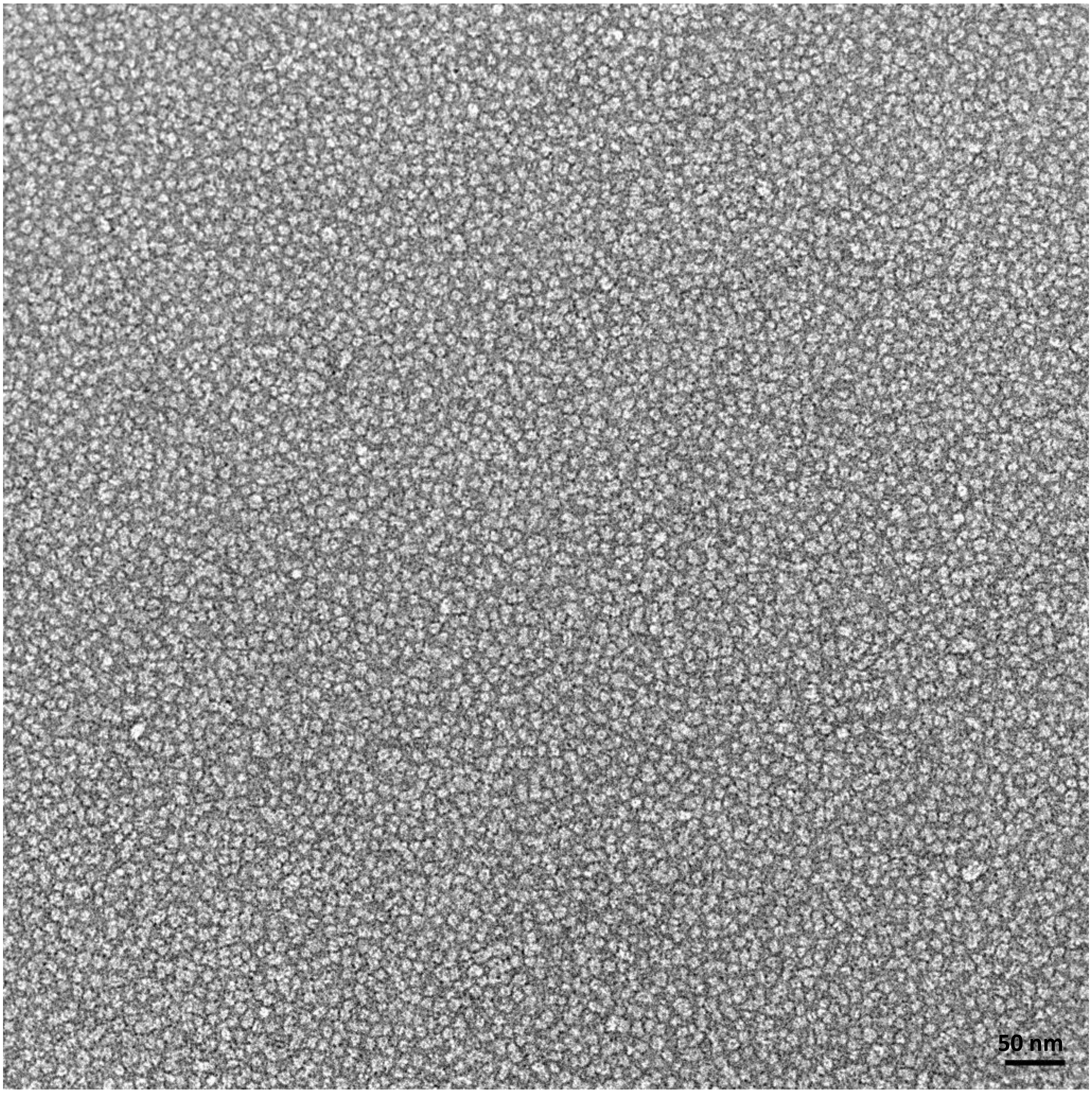

Figure S14 – Negative staining electron microscopy of AA139 titrated with ND[cNW9(POPC/POPG(4:1))] at a molar ratio of 2:1.
.

Supplemental Tables:

Table S1 - NMR data of AA139 in phosphate (PO4) buffer at pH 3.3.

	Chemical Shift (ppm)[a]																	
Residue	N	NH	Cα	Hα	Cβ	Hβ	Cγ	Cδ	Cε	Cζ	Cη	Hγ	Hδ	Hε	Hζ	Hη	Nε	Nδ2
Gly[1]			43.54	3.95														
Phe[2]	121.25	8.80	55.97	4.94	40.89	3.23		132.25	131.55	129.80			7.33	7.26	7.22			
Cys[3]	119.28	8.19	55.82	5.60	48.18	2.49, 2.86												
Trp[4]	122.88	9.26		4.85	31.88	3.31		127.34	122.81	113.91, 121.83	123.86		6.98	9.98, 7.16	7.28, 6.89	7.00	128.71	
Tyr[5]	123.12	8.69	57.57	5.09	43.56	2.94		132.66	118.01				6.90	6.76				
Val[6]	123.97	8.87	61.30	4.31		1.55	21					0.88						
Cys[7]	123.68	8.84	55.23	5.71	48.21	2.63, 3.05												
Ala[8]	125.82	9.11	50.97	4.65	22.35	1.47												
Arg[9]	120.58	8.62	55.17	5.02	27.44	1.39, 1.69	27.36					1.52	3.12	6.78				
Arg[10]	125.30	9.00	55.23	4.59	32.47	1.71, 1.79	33.48	43.4				1.54	3.21	7.22				
Asn[11]	125.26	9.58	54.23	4.42	37.51	2.80, 3.09							7.65, 6.96					112.68
Gly[12]	104.30	8.66	45.56	3.61, 4.21														
Ala[13]	123.74	7.78	50.66	4.68	21.30	1.40												
Arg[14]	122.34	8.66	56.28	4.64	30.90	1.80	28.1	43.42				1.46, 1.62	3.14					
Val[15]	127.77	8.94	61.22	4.43	34.46	2.00	20.85					0.94						
Cys[16]	123.81	8.77	55.14	5.80	48.82	2.63, 3.04												
Tyr[17]	120.80	9.04	57.38	4.93	40.45	2.93, 3.01		133.67	117.71				6.96	6.66				
Arg[18]	117.68	8.69	55.24	4.22	27.77	0.86, 1.12	31.93	40.7				1.33, 1.63	2.70, 2.83					
Arg[19]	128.47	8.69	54.23	4.30	32.62	0.62, 0.92	26.85	43.23				1.19, 1.52	3.11					
Cys[20]	117.74	8.12	55.20	5.24	48.46	2.81, 3.06												
Asn[21]	126.34	8.64	54.08	4.61		3.04, 2.92							6.59, 7.55					110.31

[a]Chemical shift are relative to DSS (0 ppm)

Table S2 – Hydrogen-deuterium exchange of AA139 peptide.

Peptide	residues with slowly exchanging amides			Hydrogen bonds
	1 h (weak)	3-4 h (medium)	>24 h (strong)	
AA139	C3, C7, R9, R10, N11, G12, R14, C16, C20, N21	A8, A13, R18	W4, V6, V15, Y17	W4-R19 (HN-O)
				V6-Y17 (HN=O)
				V15-V8 (HN-O)

Table S3 – Amide temperature coefficient of AA139.

AA139	slope (ppb/K)
Phe^{2}	-3.6
Cys^{3}	-6
Trp^{4}	-4
Tyr^{5}	-6.2
Val^{6}	-7.6
Cys^{7}	-9.2
Ala^{8}	-2.4
Arg^{9}	-7.6
Arg^{10}	-6.4
Asn^{11}	-10
Gly^{12}	-7.6
Ala^{13}	-1.4
Arg^{14}	-6
Val^{15}	-6
Cys^{16}	-9
Tyr^{17}	-2.2
Arg^{18}	-5
Arg^{19}	-5
Cys^{20}	-3
Asn^{21}	-5

Table S4 - Statistical analysis of the NMR structures of AA139. All statistics are given as mean ± S.D. Only structurally relevant restraints, as defined by CYANA, are included. Stereochemical quality parameters are those reported by Molprobity (http://molprobity.biochem.duke.edu), where the clash score is defined as the number of steric overlaps >0.4 Å per thousand atoms.

	**AA139**
**Experimental restraints[a]**	
Interproton distance restraints	
Intraresidue	66
Sequential	114
Medium range (1 < i–j < 5)	45
Long range (i–j > 5)	108
Dihedral-angle restraints (ϕ, φ)	38
Disulfide-bond restraints	12
Total number of restraints per residue	18.2
**R.m.s. deviation from mean coordinate structure (Å)**	
Backbone atoms (residues 1–21)	0.18
All heavy atoms (residues 1-21)	0.57
**Stereochemical quality[c]**	
Residues in most favoured Ramachandran region (%)	100.0 ± 0.0
Ramachandran outliers (%)	0 ± 0
Unfavourable sidechain rotamers (%)	16.2 ± 2.6
Clashscore, all atoms	0 ± 0
Overall MolProbity score	1.41 ± 0.06 (96th percentile)

Table S5. Thermodynamic parameters for AA139 and VSTx1 binding to cNDs, from individual replicates as well as the calculated average and standard error of the mean.

Peptide	K_d<br>*μM*	ΔH<br>*kcal/mol*	-TΔS[α]<br>*kcal/mol*	ΔG<br>*kcal/mol*	n
AA139	1.37	-6.94	-1.07	-8.01	4.00
	1.26	-6.19	-1.86	-8.05	4.18
	0.84	-5.73	-2.57	-8.30	4.08
Average	1.16 ± 0.28	-6.29 ± 0.61	-1.83 ± 0.75	-8.12 ± 0.16	4.09 ± 0.09
VSTx1	2.95	-2.76	-4.79	-7.55	2.0
	1.44	-1.53	-6.44	-7.97	2.2
	1.68	-1.48	-6.4	-7.88	1.8
Average	2.02 ± 0.81	-1.92 ± 0.73	-5.88 ± 0.94	-7.80 ± 0.22	2.00 ± 0.20
